# Epigenetic and Conventional Regulation Is Distributed among Activators of *FLO11* Allowing Tuning of Population-Level Heterogeneity in Its Expression

**DOI:** 10.1371/journal.pgen.1000673

**Published:** 2009-10-02

**Authors:** Leah M. Octavio, Kamil Gedeon, Narendra Maheshri

**Affiliations:** 1 Computational and Systems Biology Initiative, Massachusetts Institute of Technology, Cambridge, Massachusetts, United States of America; 2 Department of Chemical Engineering, Massachusetts Institute of Technology, Cambridge, Massachusetts, United States of America; European Molecular Biology Laboratory, Germany

## Abstract

Epigenetic switches encode their state information either locally, often via covalent modification of DNA or histones, or globally, usually in the level of a *trans*-regulatory factor. Here we examine how the regulation of *cis*-encoded epigenetic switches controls the extent of heterogeneity in gene expression, which is ultimately tied to phenotypic diversity in a population. We show that two copies of the *FLO11* locus in *Saccharomyces cerevisiae* switch between a silenced and competent promoter state in a random and independent fashion, implying that the molecular event leading to the transition occurs locally at the promoter, in *cis*. We further quantify the effect of *trans* regulators both on the slow epigenetic transitions between a silenced and competent promoter state and on the fast promoter transitions associated with conventional regulation of *FLO11*. We find different classes of regulators affect epigenetic, conventional, or both forms of regulation. Distributing kinetic control of epigenetic silencing and conventional gene activation offers cells flexibility in shaping the distribution of gene expression and phenotype within a population.

## Author Summary

In an uncertain and changing world, microbial populations with a diverse range of phenotypes may outperform a monolithic population. Over many generations, mutations can lead to genetic diversity in a population. However, microbes have strategies to generate such diversity quickly. For example, if multiple genes switch ON and OFF slowly, randomly, and independently of each other, then a large combination of gene expression states, and hence phenotypes, are possible. The different gene expression states do not involve changes in DNA sequence and are therefore epigenetically inherited. We show that the two copies of the *FLO11* gene in *S. cerevisiae* can switch ON and OFF slowly and independently. In addition, we reveal a simple regulatory strategy by which cells can control the proportion of cells in different gene expression states. Because *FLO11* encodes a cell-wall protein responsible for mediating cell–cell and cell–surface interactions, this control might literally allow natural populations to have a controllable fraction of cells “stick around” while the other fraction is easily washed away.

## Introduction

Microbial cell populations employ a number of strategies to rapidly generate phenotypic diversity on relatively short time scales [1],[2]. In some microbes, genes known as contingency loci contain tandem repeats of DNA whose recombination results in turning expression ON or OFF [3]. Other genetic strategies include the directed recombination of silent alleles into a particular active locus, as is the case for mating type switching in yeasts and surface antigen expression in T. *brucei* [4], the causative agent of African sleeping sickness. Another widely used strategy that generates phenotypic heterogeneity in clonal microbial cell populations is epigenetic gene regulation. In contrast to genetic strategies, this refers to the heritable change in a gene's expression that is not caused by changes in the underlying gene sequence. For example, the parasite *P. falciparum* (malaria) and the model organisms *S. cerevisiae* and *E. coli* use epigenetic mechanisms to variably express antigenic cell-surface proteins [2] and possibly escape immune surveillance and/or survive in an unpredictably changing environment.

Many epigenetically regulated genes can be considered switches as they have two heritable expression states, “ON” and “OFF.” A stable epigenetic marker maintains each state and can be encoded in *cis* or in *trans*. The molecular basis of local, *cis* markers involve covalent modifications of DNA or DNA-associated proteins. These include DNA methylation [5] and histone modifications that define silenced heterochromatin or active euchromatin in eukaryotes [6]. Global, *trans* markers are often transcription factor activity; the mechanism for stable, slow switching of these levels is positive or double negative feedback loops that generate heritable bistable gene expression states associated with high or low levels of transcription factor activity [7]–[9]. Switches using either scheme respond to environmental factors, but heterogeneity is observed even with constant environmental conditions, suggesting that the switch can rarely and randomly be toggled due to fluctuations in the intracellular environment. The two schemes can be also combined. For example, in uropathogenic *E. coli* the expression of pyelonephritis-associated pili is regulated by an epigenetic switch that maintains its state through both DNA methylation and a positive feedback loop [10].

The control of phenotypic heterogeneity is arguably as important as its rapid generation. Heterogeneity, or noise, in conventionally regulated gene expression has been well-studied in recent years. Single cell and single molecule studies have revealed that gene activation occurs in random, intermittent transcriptional bursts [11]–[14] due to fast promoter fluctuations (>once per cell cycle) between an inactive (but competent) and active promoter state. Mechanistically, this is an oversimplification as the promoter likely adopts a series of different states involving binding of various gene-specific and general transcriptional machinery that lead to productive transcription. Here, the active promoter state can be thought of as one where rapid initiation and reinitiation is possible. For example, for regulable RNA Pol II–dependent promoters, transcriptional initiation is often rate-limiting and hence the active promoter state corresponds to pre-initiation complex formation. Expression heterogeneity caused by even these fast fluctuations can have consequences on phenotype and population-level fitness [15].

Noise in gene expression can be partitioned depending on whether its source is intrinsic or extrinsic to the process of gene expression. Intrinsic noise is due to the random nature of chemical transformations, including transcription and translation events. However, the random bursts of transcription thought to be associated with fast promoter fluctuations occurring in *cis* appear to be the dominant source of intrinsic noise in eukaryotes [16],[17]. Extrinsic noise is due to cell-to-cell variation in *trans* factors affecting gene expression: for example, general and gene-specific transcriptional machinery, ribosome number and tRNA availability, or even cell morphology. The two sources can be experimentally distinguished using a dual-reporter assay, where two copies of the same promoter are used to drive distinguishable fluorescent protein variants [18]. Extrinsic noise is variation in protein levels between different cells; intrinsic noise is variation in protein levels within the same cell.

How regulators control the kinetics of intrinsic promoter fluctuations dictates the resulting expression heterogeneity. Stochastic models can be used to directly quantify this relationship [19]. Most transcriptional regulators appear to function by modulating the frequency of these bursts [19],[20], probably in large part by increasing the rate of transcriptional initiation. Therefore, regulators do not control expression heterogeneity independently of expression level. In fact, heterogeneity is under genetic control as noisy promoters tend to have particular characteristics: strong TATA boxes, highly regulable, and dependent on chromatin remodeling activities [19]–[21].

While conventional gene regulation involves fast fluctuations between inactive (competent) and active promoter states, epigenetic silencing of gene expression involves slow fluctuations (<once per cell cycle) between a silenced and competent state. The kinetics of these fluctuations in *trans*-encoded switches involving feedback loops and associated with bistable gene expression have been studied in detail [7],[22],[23]. Both theory and experiment suggest that extrinsic fluctuations in the *trans* factor that overcome the stability of the two epigenetic states lead to switching [7]. However, much less is known of the precise role of regulators in modulating fluctuations of *cis*-encoded switches which must involve changes in the local promoter state. For example, activators could increase population-averaged expression by either stabilizing the competent state or destabilizing the silenced state. The resulting heterogeneity in expression is dictated by the specific kinetic role of the activator.

In a diploid organism, an epigenetically regulated gene might exhibit four different expression states if each copy switches independently. With global encoding, both copies respond to the same global factor and must switch in a correlated manner. However, with local encoding, each copy may respond independently if the fluctuation that trips the switch is a molecular event that occurs locally at one copy. In fact, a recent study demonstrated the random and independent switching of two copies of a reporter gene inserted within the canonically silenced mating type loci, *HMR* and *HML*, in *S. cerevisiae*. Four distinct expression states were observed in a *sir1* background, where SIR-protein dependent silencing of these loci is partially impaired [24].

Multiple *cis*-encoded epigenetic switches that toggle slowly and randomly could lead a combinatorial explosion of expression states and represent a powerful strategy to generate phenotypic diversity. Is independent switching employed in nature and how are slow fluctuations regulated? The *S. cerevisiae* Flo11p is a cell-wall adhesin protein and member of the *FLO* gene family important in mediating cell-to-cell and hydrophobic cell-surface interactions [25]. In addition to traditional regulation via the MAPK and PKA pathways [26],[27], at least three mechanisms are known to generate variation in cell-surface adhesins: ploidy regulation [28], frequent recombination of tandem repeats within adhesin genes [29] and epigenetic silencing [30]. Silencing at *FLO11* occurs in a SIR-protein independent manner and is both promoter and position-specific [30]. Given the importance of phenotypic diversity in the adhesive phenotype and the epigenetic silencing at *FLO11*, independent switching could represent a fourth mechanism for generating variation.

**Figure 1:**
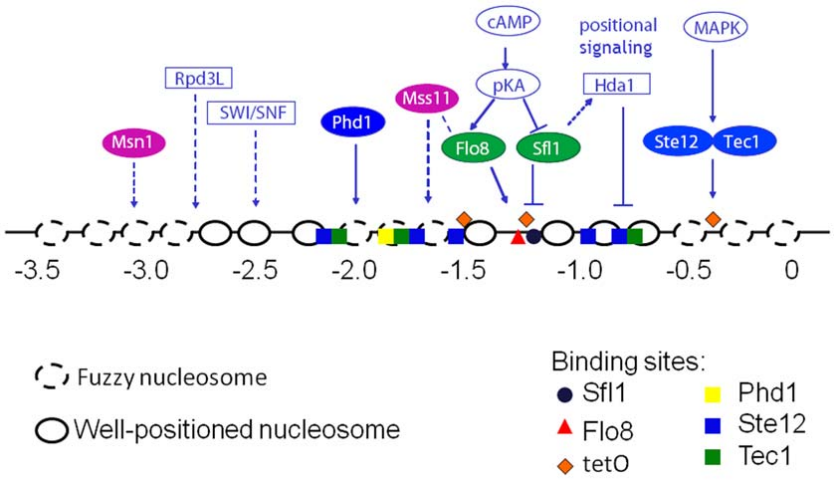
Signals from many *trans* factors converge at the complex *FLO11* promoter. Regulators of *FLO11* transcription. Nucleosomal positions are based on a thermodynamic model for nucleosomal occupancy [37]. Binding sites are approximate and based on literature but most sites have not been confirmed directly. The three locations where a tetO sequence was inserted are also shown. See main text, Text S1, and Figure S1 for further details.

At 3.5 kb, the *FLO11* promoter is one of the largest in *S. cerevisiae* and regulated by many factors (Figure 1) whose kinetic roles are unknown. Silencing of *FLO11* is thought to occur through the recruitment of the histone deacetylase Hda1p via the repressor Sfl1p through a yet to be defined mechanism [30]. The Sfl1p repressor binding site overlaps the Flo8p activator binding site [27]. Activation of *FLO11* through the protein kinase A (PKA) pathway results in phosphorylation of both Sfl1p and Flo8p. While phosphorylation disables Sfl1p binding, it enables Flo8p binding [26],[27]. Additional transcription factors bind directly to this promoter [26],[27],[31] including the MAPK regulated Ste12p/Tec1p and Phd1p. These three activators require Flo8p for activation and play a significant role in determining the overall level of expression [32]. Two activators, Msn1p and Mss11p, do not require Flo8p for activation and operate through poorly understood mechanisms that do not seem to require DNA binding [33]. Msn1p acts at longer distances to destabilize chromatin [34]; Mss11p has glutamine rich activation domains and may associate weakly with Flo8p [32]. All these activators modulate plasmid-borne *FLO11* expression, a context where silencing does not occur [26],[27]. However, their varied biochemical roles might imply distinct kinetic and functional roles in epigenetic regulation of *FLO11*.

Here, we provide evidence that *FLO11* is indeed a *cis*-encoded epigenetic switch and identify the kinetic roles of *trans* factors in the epigenetic and conventional regulation of *FLO11*. Within a diploid yeast, each locus switches in a slow, random, and independent manner, with switching rates dependent on environmental conditions. Using a stochastic kinetic model, we infer the kinetic role that different regulators have on the slow promoter fluctuations associated with epigenetic transitions between a silenced and competent promoter state and the fast promoter fluctuations associated with conventional gene activation. We find three classes of *FLO11* regulators: those that affect the stability of the competent state, affecting slow promoter fluctuations; those that regulate the burst frequency of transcription due to fast promoter fluctuations; and those that have both functions. Moreover, a single synthetic activator can mimic each of these three classes based on the location of its DNA binding site. Because the kinetic role of each regulator defines its impact on expression heterogeneity, this can be controlled by the choice of regulator class. Finally, ethanol controls the extent of gene silencing nearly independently of transcriptional activation through Flo8p, thereby dictating whether *FLO11* expression responds in a graded or heterogeneous manner to other signals.

## Results

### *FLO11* switches between silenced and competent states independently at each locus

**Figure 2:**
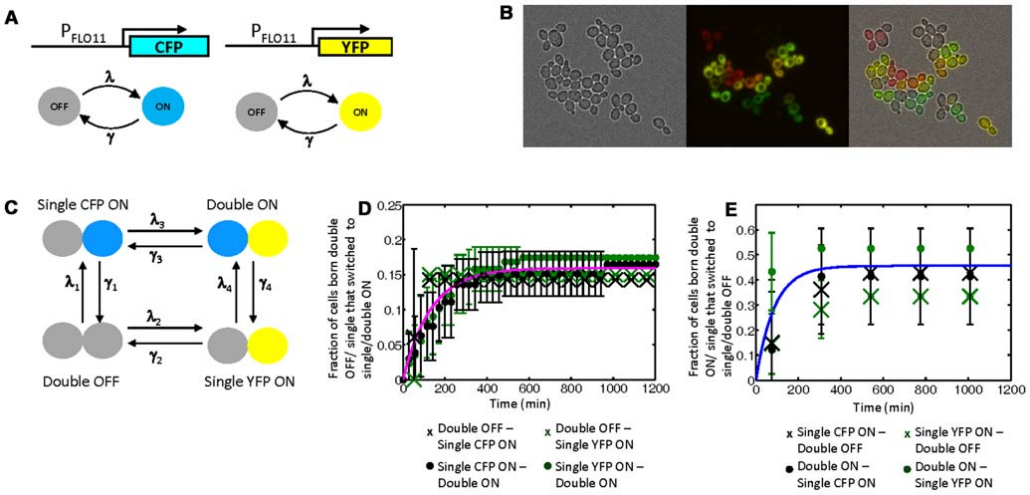
Mixed expression states and independent switching at the *FLO11* locus. (A) Dual reporter assay. Each *FLO11* allele turns ON and OFF slowly with identical rates *λ* and *γ* because the two reporters are equivalent. (B) Mixed expression states. A dual reporter strain grown in rich media (no glucose) supplemented with 1% ethanol and 2% glycerol (false color overlay CFP = red, YFP = Green). All four possible expression states are seen. (C) Transition rates. Equivalence of reporters implies *λ*_1_ = *λ*_2_, *λ*_3_ = *λ*_4_, *γ*_1_ = *γ*_2_, *γ*_3_ = *γ*_4_. Independent switching implies *λ*_1_ = *λ*_3_ and *γ*_1_ = *γ*_3_. (D) OFF to ON transition rates of different expression states: *λ*_1_ (X), *λ*_2_ (X), *λ*_3_ (•), *λ*_4_ (•). Each marker represents the fraction of cells observed to switch at the particular time, and the pink curve is the same as the fit curve in Figure 3A. (E) As in (D) but for ON to OFF transition rates: *γ*_1_ (X), *γ*_2_ (X), *γ*_3_ (•), *γ*_4_ (•). The blue curve is the same as the fit curve in Figure 3A. (D) and (E) demonstrate that transition rates at one allele are independent of the state of the other allele. Even the null hypothesis that *γ*_2_ and *γ*_4_ are equivalent cannot be rejected at the 5% significance level (two-way T-test, p = 0.28) nor can the null hypothesis that their distributions are identical (two-way KS test, p = 0.47).

Under poor nutritional conditions, *FLO11* is partially silenced in haploid cells and heterogeneous in expression. Members of this population are capable of reversibly transitioning between the OFF (silenced) and ON (competent) state [30]. To determine whether the transitions were due to a *cis* or *trans* fluctuation, we employed the dual-reporter assay, replacing the two copies of a *FLO11* ORF in diploid yeast with a distinct fluorescent protein variant (Venus YFP and Cerulean CFP) (Figure 2A). Importantly, we verified the independence and equivalence of the two reporters with respect to the presence of the other reporter (Figure S2 and Figure S3). When grown in media with poor carbon sources, including ethanol, glycerol, galactose, and raffinose, we observed all four possible expression states (Figure 2B, data not shown). Because endogenous Flo11p is not present in the dual-reporter strain, we verified that Flo11p did not affect expression at the *FLO11* promoter in two ways. First, we added a plasmid constitutively expressing *FLO11* and found no significant effect on fluorescent protein expression (data not shown). Second, we compared fluorescent protein expression in the dual-reporter strain to strains where only one *FLO11* allele had been replaced with a fluorescent protein. There was no difference in expression levels (Figure S2 and Figure S3).

**Figure 3:**
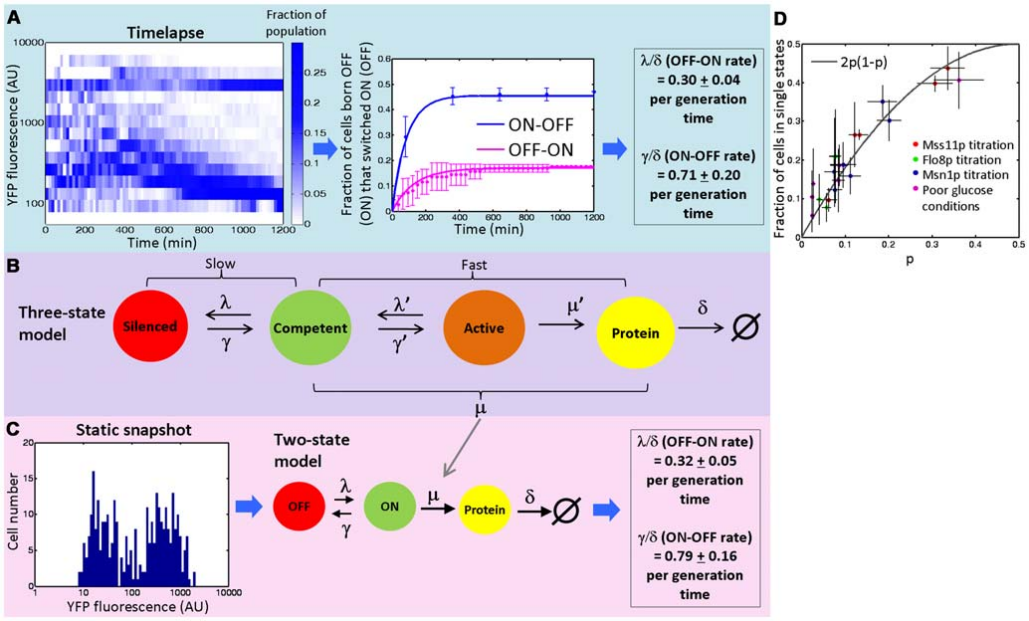
A method for inferring kinetics of switching from static steady state distribution. (A) (Left) Time evolution of the population distribution of YFP expression from a dual-reporter strain growing in YP 1% ethanol, 2% glycerol within a microfluidic chamber over 20 hours. Colorbar indicates fraction of cells (*n* = 230 over time course). This strain had been growing in identical conditions in liquid culture prior to transfer to the microfluidic chamber. The distribution changes early on because of the small initial sample size (*n* = 10). (Right) Marginal transition rates between ON and OFF states. Blue/pink dots indicate fraction of cells ON/OFF at birth and observed to switch OFF/ON. Corresponding curves are fits of the model for exponentially distributed switching times from ON to OFF and OFF to ON, with adjusted rates shown next to the plot. Error bars correspond to 3 s.d. from the mean calculated by a bootstrap analysis. Similar results are obtained when focusing on CFP expression (see Figure S5 and Figure S6). (B) Three-state model of *FLO11* activation showing separation of timescales between epigenetic (silencing) and conventional regulation. When slow transitions associated with silencing are present, the fast transitions of transcriptional bursting can be lumped into a single rate μ. The model then collapses into the two-state model in (C). (C) (Left) Static distribution of YFP fluorescence of dual-reporter strain grown in identical media conditions as A but in deep well plates rather than the microfluidic device. Transition rates inferred from this snapshot using a stochastic kinetic model (right) agree closely with those obtained by timelapse microscopy. (D) Modulation of switching rates. The stochastic kinetic model's prediction of the fraction of cells in the mixed expression state corresponds to independent switching (given by 2*p(1−p)*, corresponding to the gray line) for a range of conditions. Error bars (x-axis) are from 95% confidence intervals from MLE fit of switching rate to estimate true fraction of ON cells; error bars (y-axis) are due to errors in the estimation of threshold fluorescence value for autofluorescence (see Text S1).

If each allele switches independently, then at steady-state the proportion of cells in each expression state is given by *p^2^* (both ON), (1−*p*)^2^ (both OFF), or 2*p*(1−*p*) (mixed ON/OFF and OFF/ON), where *p* is the proportion of cells with a particular allele ON. Note that *p* is identical for both YFP and CFP expression because the alleles are equivalent. We were able to verify the population's expression profile had reached steady-state (Figure 3A and Figure S4). However, a naïve classification of expression state based on comparing a cell's fluorescence level to background is incorrect because it does not consider the long lifetime of the fluorescent proteins which obscures the true expression state of the promoter. Therefore, we directly measured the eight transition rates by real-time monitoring of *FLO11* expression in single cells grown in a microfluidic chamber at constant conditions (Text S1 for details). All four ON to OFF and OFF to ON transition rates (Figure 2C–2E) were found to be indistinguishable, demonstrating that each allele was switching independently. Furthermore, the fraction of cells turning ON or OFF are well-fit by a single exponential, confirming each transition is appropriately lumped as a pseudo-first order reaction. Switching was uncorrelated with cell-cycle stage (Figure S7).

### Using a stochastic kinetic model, static distributions can reveal kinetic information

Time lapse microscopy provides an accurate determination of the slow epigenetic transition rates and proportion of each expression state, but it is experimentally challenging and low throughput. Therefore, after determining that transition rates were accurately described as first order, we devised a way to infer these rates directly from static snapshots, accounting for the long lifetimes of the fluorescent reporters. Two-state models have been widely employed to model faster promoter fluctuations associated with conventional gene regulation [16],[21],[35]. In such models, the promoter can transition between an inactive but competent state and an active state that leads to transcription. Many eukaryotic genes appear to reside in the competent state, with rare transitions to the short-lived active state that result in a “burst” of transcription.

The observed variation in the *FLO11* promoter can be divided into an intrinsic and extrinsic component. The *FLO11* promoter is subject to both fast intrinsic fluctuations and a slow epigenetic transition, as depicted by the augmented three-state model in Figure 3B. Extrinsic noise also contributes to cell-to-cell variation in *FLO11* expression levels when the promoter is not silenced. However, when the promoter is (partially) silenced, the predominant source of variation in *FLO11* or reporter expression arises from the slow epigenetic transition between silenced to competent states because of (1) the smaller magnitude of the fast intrinsic and extrinsic fluctuations and the fact that (2) the faster fluctuations (<1 cell generation) are more completely time-averaged by the long-lived reporter compared to the slow transition (>1 cell generation). Therefore, we can lump the fast transition rates (*λ*′, *γ*′, *μ*′) into an overall transcription rate *μ* and ignore extrinsic fluctuations. Gene expression can now be described using the commonly employed two-state model:
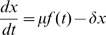
, where *x* i*s* the amount of reporter protein, *μ* is the (lumped) protein production rate, *δ* is a protein degradation rate (here the cell growth rate), and *f(t)* is a “random telegraph process” that takes values of 0 or 1 corresponding to a silenced or active promoter state, with exponentially distributed times between switching events (Figure 3C). This stochastic equation has been solved analytically to yield a Beta distribution for protein number *x* at steady-state [35]. The slow epigenetic transition rates, *λ* and *γ*, correspond to those measured in the time lapse experiment. To infer these rates we assume our measured distribution of protein *x* is steady (Figure S4) and fit it to the Beta distribution using a value of *μ* based on the expression level of the ON population in a bimodal condition (the parameter *δ*, the cell growth rate, is measured directly - see Text S1).

We tested this method in two different ways. First, we used the steady-state protein distribution of the time lapse experiments to estimate transition rates and found tight agreement between the inferred rates and those directly measured in time lapse (Figure 3A and 3C). Second, this model allows proper estimation of the fraction of cells that appear ON in static distributions that are actually OFF because of the long lifetime of the fluorescent reporter (details in Text S1). We applied this correction to static snapshots of cells grown in different conditions. Although the fraction of cells in each expression state varied, the overall statistics were always consistent with independent switching at each promoter (Figure 3D). Therefore, the upstream signaling network can map environmental inputs to a particular mixture of expression states through the modulation of transition rates.

### A strategy for determining how regulators affect transition rates

Ultimately, environmental signals modulate epigenetic regulation of *FLO11* through downstream regulators. The effect of these regulators on both the mean level of expression and expression heterogeneity is succinctly and quantitatively described by their effect on the transition rates in the three-state model (Figure 3B). Therefore, we decided to titrate *trans* factors and measure the quantitative response of the *FLO11* promoter at the single cell level in hundreds of cells by fluorescence microscopy using the dual-reporter assay. For each condition and strain, we always grew cells for >10 doublings, serially diluting them as needed to maintain low density and ensure a steady-state had been reached (further details in Text S1).

**Figure 4:**
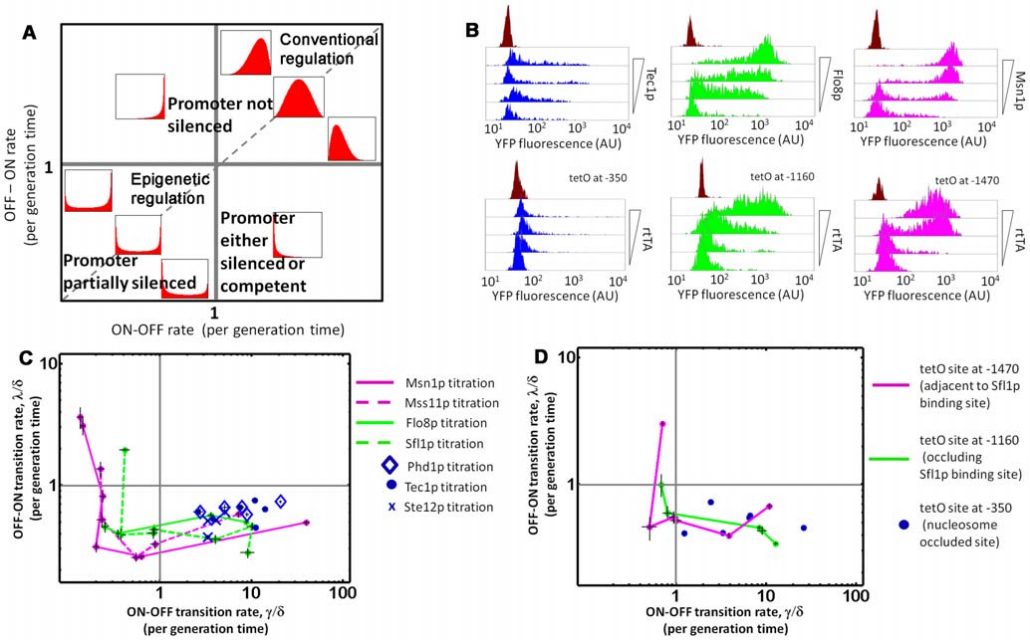
Three different kinetic roles for regulators of *FLO11*. (A) The qualitative shape of the Beta distribution for various values of OFF→ON (*λ*/*δ*) and ON→OFF (*γ*/*δ*) transition rates (normalized with respect to the growth rate *δ*). When both rates are slower than growth (lower left quadrant) they characterize slow epigenetic transitions between the silenced and competent states. The expression distribution is bimodal, representing stable ON and OFF populations. These rates can be inferred by measuring the expression distribution by fluorescence microscopy and fitting to the Beta distribution. For unimodal ON distributions, epigenetic silencing no longer occurs. If only fast intrinsic fluctuations between the competent and active promoter state were present, the same two-state model would apply, but now predict the fast transition rates and unimodal distributions (upper half of plot). However, because extrinsic fluctuations also matter, direct fitting of measured unimodal distributions does *not* yield the fast transition rates (see main text and Text S1 for details). (B) Representative fluorescence histograms of the three activator classes. (Top) Tec1p titrated in wildtype background in SD ura-; Flo8p titrated in *flo8Δ* background in SD ura- +1% ethanol; Msn1p titrated in wildtype background in SD ura-. (Bottom) rtTA titrated in strain with tetO at −350 (nucleosome occluded site), at −1160 (site occludes Sfl1p binding site), and at −1470 (site directly upstream of the −1200 nucleosome free region). Histograms are derived from fluorescence microscopy (cell number >300). The fluorescence distribution of an OFF strain (Y92) used to measure autofluorescence is shown at the top of each plot. (C) Kinetic roles of regulators. Increasing levels of various activators of *FLO11* decrease *γ*, stabilizing the active state without significantly changing *λ*. Class I activators cannot decrease *γ* significantly (blue). Class II activators can shift the transition rates into the lower left quadrant which corresponds to partially silenced, bimodal expression (pink). Flo8p has a less stable silenced state compared to the class II activators. It appears that at a critical value of *γ* the regulators abolish silencing, and the response enters the upper left quadrant. (D) Synthetic activator titration. Titrations of synthetic activators mimic the three classes of activators, depending on the location of the binding site. All titrations (B, C, and D) were in SD ura- except Sfl1p and Flo8p where 1% ethanol was added. Error bars represent 95% confidence intervals (to fits of experimental data to the Beta distribution for a single value of *μ*—details in Text S1).

To obtain transition rates, we fit the measured fluorescence distributions arising from each titration to the Beta distribution, the solution to the simplified two-state model. As described previously, a two-state model which lumps the fast transitions is only strictly applicable when slow epigenetic transitions related to silencing dominate. The four quadrant plot in Figure 4A summarizes the qualitative population-level response as given by the Beta distribution for various combinations of *λ* and *γ*. Each quadrant corresponds to regimes where *λ* and *γ* are slower or faster than the division rate (*δ*). Epigenetic regulation occurs by definition in the lower left quadrant, when both *λ* and *γ* are slower than the division rate (*λ∼δ* and *γ∼δ*<1). Expression can turn completely OFF if the active state is destabilized (*γ* increases, shift to lower right quadrant), or the silenced state is stabilized (*λ* decreases, bimodal expression with vanishingly smaller percentage of cells ON). Opposite changes in *λ* and *γ* turn expression completely ON (and can lead to a shift to the upper right quadrant).

If epigenetic regulation is lost, expression levels can still change due to faster promoter fluctuations. A two-state model accurately describes intrinsic (but not extrinsic) fluctuations caused by transitions between the competent and active promoter states. For example, a conventionally regulated (but repressed) gene can be OFF and lie in the lower right quadrant. Activation leads to an increased burst frequency (*λ′* increases) and the graded, unimodal distribution of the upper right quadrant. Importantly, it is impossible to distinguish between conventional repression and epigenetic silencing in any population in the lower right quadrant where expression is completely OFF. Furthermore, fitting fluorescence expression distributions generated from a single *FLO11* promoter driven reporter that is conventionally regulated does not yield the fast promoter transition rates *λ′* and *γ′* because here extrinsic fluctuations are significant. The extrinsic noise is due to cell-to-cell variation in factors like morphology, ribosome number, and/or upstream components in the *FLO11* regulatory pathway and affects both promoters within the same cell in a correlated fashion. To properly measure the fast transition rates associated with conventional regulation, the intrinsic noise should be analyzed to determine the burst frequency (*λ′*) and burst size (*μ′*/*γ′*) (see Text S1).

### Three classes of regulators of FLO11 expression

To decouple complex upstream signaling events occurring at the promoter (Figure 1), individual *trans* factors were expressed heterologously under the control of a doxycycline-inducible promoter [36]. Because Sfl1p and Flo8p are post-translationally regulated, we needed a way to tune their relative strength. We chose ethanol, since the addition of ethanol activates *FLO11* expression in a Flo8p-dependent manner (see below). All the *trans* factor titrations were performed in either SD ura- or SD ura- with ethanol. Titrations of Sfl1p, Flo8p, and Mss11p were done in a *sfl1Δ*, *flo8Δ* or *mss11Δ* background, respectively.

For each titration point, we inferred the transition rates *λ* and *γ* using the two-state model. To summarize the effect of various activators and Sfl1p on the stability of the silenced and competent states, we plot the series of (*γ*,*λ*) values determined on the four quadrant plot of Figure 4A. In SD ura-, *FLO11* is OFF, corresponding to the lower right quadrant. Based on the response of the *FLO11* promoter (Figure 4B and 4C), we grouped the activators into 3 classes. Addition of three Class I activators, Tec1p, Ste12p, and Phd1p, appears to weakly stabilize the competent state, but expression remains extremely low. The Class II activators, Msn1p and Mss11p, stabilized the competent state by decreasing *γ* and entering the heterogeneous region where slow promoter fluctuations dominate. At some critical *γ* value, the silenced state is rapidly destabilized and the entire population turns ON. Flo8p constitutes a special class and was titrated in ethanol conditions where presumably some fraction of Flo8p is now phosphorylated and active. The population response is intermediate between Class I and Class II activators, but closer to Class II. Sfl1p has the exact opposite effect under the same ethanol conditions, consistent with the antagonistic role Sfl1p and Flo8p have through their overlapping binding sites and as being negatively and positively regulated by PKA, respectively.

To determine if activator class was correlated to binding site position or accessibility, we applied both an *in silico* nucleosomal occupancy model [37] and performed micrococcal nuclease mapping (Figure 1 and Figure S8) of the *FLO11* promoter. Both techniques suggested the −1200 region containing overlapping binding sites for Sfl1p and Flo8p is nucleosome free. In contrast, binding sites of Class I activators occur in nucleosomally occluded regions. Class II activators are not known to bind DNA but are potent activators, with Msn1p having a known ability to recruit chromatin remodeling machinery [34].

### A synthetic activator mimics each activator class depending on its binding site position

Are binding site position, accessibility and/or competition with Sfl1p sufficient to determine activator class? If so, a synthetic activator could have qualitatively different regulatory profiles depending on binding site position. We engineered dual-reporter yeast strains with the 19 bp tetO sequence inserted at 3 different locations within the *FLO11* promoter. The first location was at −350 in a nucleosomally occluded region close to the TATA box and transcriptional start site. The second location was at −1470, on the outer-edge of the nucleosome upstream of the Sfl1p binding site and far from the core promoter. The third location was at −1160, within a nucleosome-free region directly overlapping the Sfl1p binding site. Sfl1p binds as a dimer at two sites [27],[38], so we replaced 19 bp of promoter sequence between the two sites with the tetO sequence to preserve the distance spanned by the two sites. The tetO is bound by rtTA, a synthetic activator that contains a strong acidic activation domain, VP16 [36], known to recruit the SAGA complex in yeast [39]. We titrated rtTA in these three strains grown in SD ura- (all initially OFF). Each location functionally mimics the response of the respective class of activators (Figure 4B and 4D). Importantly, the silenced state stability is reduced for the third tetO location compared to the second tetO location. This occurred even in the absence of ethanol, suggesting that the difference in silenced state stability between Class II activators and Flo8p is not due to an alternative ethanol-specific effect.

### Two modes of Sfl1p repression correspond to a graded or heterogeneous response

**Figure 5:**
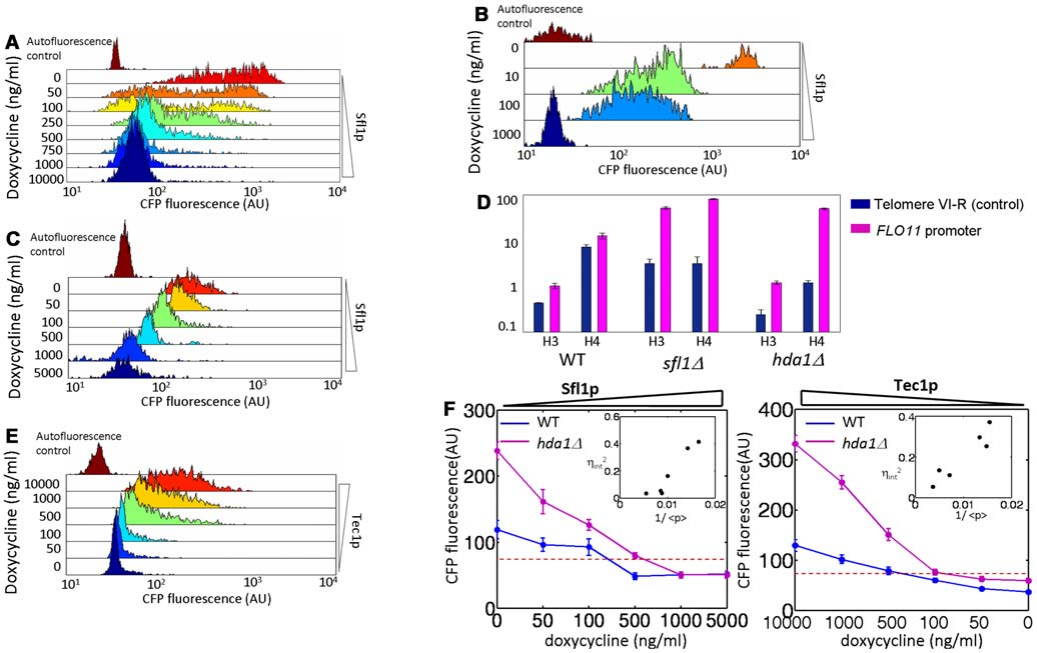
Hda1p is necessary for silencing and a heterogeneous response. (A) Sfl1p titrated in *sfl1Δ* background in SD ura- +1% ethanol leads to a heterogeneous response. (B) Sfl1p titrated in *sfl1Δ* background in SD ura- leads to a graded response. (C) Sfl1p titrated in *hda1Δ* background in SD ura- +0.5% ethanol reverts to a graded response. Expression is lower with no doxycycline because of endogenous Sfl1p expression. (D) ChIP assay probing for acetylated H3 and H4 histones at *FLO11* promoter (strains grown in SD complete or SD leu-). Probes amplified the −1.7 to −1.5 kb region of the promoter. Signal (y-axis) represents anti-acetylated histone/anti-histone ratio, or an effective average acetylation per histone in the region. Deletion of both *sfl1* and *hda1* result in hyperacetylation of the *FLO11* promoter which is associated with the abrogation of silencing. Therefore *SFL1*-dependent silencing at *FLO11* requires *HDA1*. Error bars are standard error of triplicate quantitative PCR samples. (E) When the activator Tec1p is titrated in an *hda1Δ* background, the response is also graded (SD ura-). (F) Mean levels of expression during Sfl1p and Tec1p titrations in wildtype (blue curve) and *hda1Δ* (pink curve) backgrounds. Elimination of silencing because of lack of Hda1p lowers the threshold level at which activators function. Furthermore, the population response is graded (C and E, see Figure S8 for other activator titrations). Error bars represent 3 s.d. around mean calculated from bootstrap analysis. Inset: The square of intrinsic noise of Sfl1p (left) and Tec1p (right) titrated in *hda1Δ* is proportional to the reciprocal of protein abundance (here shown as the mean fluorescence level). This indicates that without silencing, regulators modulate expression by controlling burst frequency (*λ'*).

When performing the Sfl1p titration above (Figure 4C and Figure 5A), we did so in a *sfl1Δ* background in ethanol. *FLO11* was highly expressed (upper left quadrant) in the *sfl1Δ* background, as has been shown previously [26]. Surprisingly, in the Sfl1p titration in SD ura- media without ethanol, both promoters turned off in a *graded* fashion (Figure 5B). This was in contrast to the heterogeneous population response observed for titrations of Class II activators (Figure 4) including Sfl1p titrations performed in ethanol (Figure 5A), a condition where Flo8p is presumably more active. To explain this result, we hypothesized that a critical Sfl1p level is required to silence the promoter, and below this level Sfl1p still repressed transcription but in a conventional manner associated with faster promoter fluctuations. The model requires that Sfl1p is able to repress the *FLO11* promoter as a silencer or as a conventional repressor; evidence exists for both modes [38],[40].

To test this model, we generated a dual-reporter strain in an *hda1Δ* background and added back Sfl1p heterologously. When we titrated Sfl1p in this background in SD ura- media, we observed a graded response (Figure 5C). This establishes that Sfl1p is capable of repressing expression in an Hda1p-independent manner. The graded response suggests Sfl1p is working as a conventional repressor rather than affecting the slower fluctuations between the epigenetically silenced and competent promoter states. To further demonstrate that Hda1p is necessary to silence *FLO11*, we measured the average H3 and H4 histone acetylation state at the *FLO11* promoter by chromatin-IP in the dual-reporter strain grown in SD ura- (Figure 5D). Only the wildtype *FLO11* promoter exhibited a hypoacetylated state, indicative of silenced chromatin [41] and similar to a silenced telomeric region. In both the *hda1Δ* and *sfl1Δ* backgrounds, the promoter was hyperacetylated, a chromatin state associated with lack of silencing. Together, this demonstrates that Sfl1p silences the promoter in an Hda1p-dependent manner and the silenced state at *FLO11* is correlated with hypoacetylation in at least one region (∼−1600 bp) of the promoter.

If silencing is eliminated in an *hda1Δ* background, titration of activators in the presence of high levels of Sfl1p will result in a graded response, since Sfl1p is now functioning as a conventional repressor. In addition, the threshold level of activator required to turn on *FLO11* will be lower. We performed these titrations (shown for Tec1p in Figure 5E and 5F and other activators in Figure S8) and confirmed this prediction. In addition, the intrinsic noise of these strains was proportional to the inverse square root of the mean expression level (Figure 5F inset and Figure S8), indicating that without slow promoter fluctuations, the activators regulate the burst frequency, *λ′* [19].

### Ethanol controls the importance of silencing in a Flo8p-dependent manner

**Figure 6:**
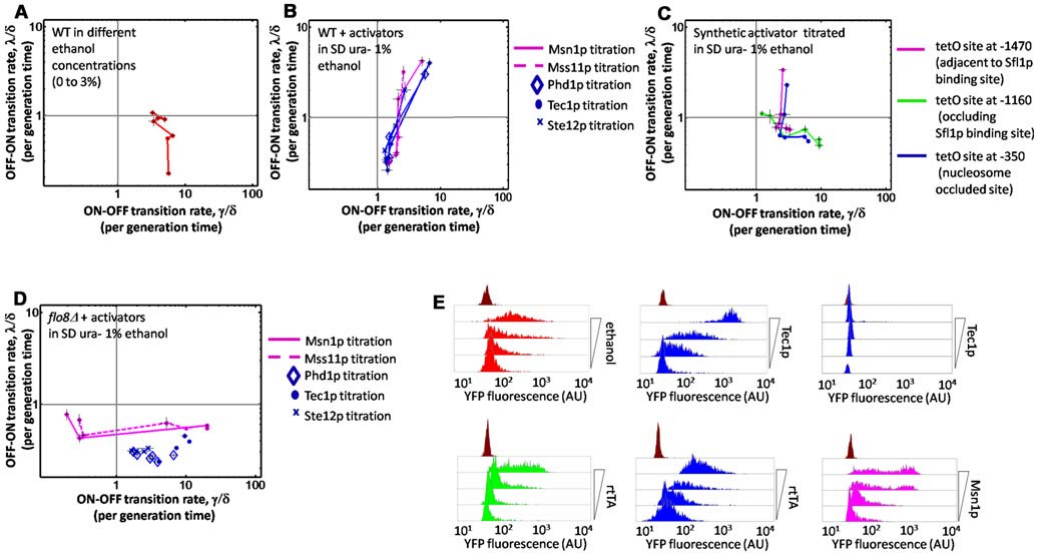
Ethanol modulates silencing at the promoter via Flo8p. (A) Wildtype grown in SD complete with ethanol added to final concentration ranging between 0 and 3%. (B) Activators titrated in wildtype background in SD ura- +1% ethanol. All responses are graded, suggesting loss of silencing at the promoter. (C) Synthetic activator (rtTA) titration in SD ura- +1% ethanol. As in (B), responses are also graded. (D) Activators titrated in *flo8Δ* in SD ura- +1% ethanol. The response is closer to that in Figure 4A rather than Figure 6B, indicating that ethanol abolishes silencing at the promoter through Flo8p. (E) Representative fluorescence histograms of titrations shown in (A, B, C, D). (Top) Wildtype titrated as in panel A, Tec1p titrated as in (B), and as in (D). (Bottom) rtTA titrated in strain with tetO site at −1160 (occluding Sfl1p binding site), and at −350 (nucleosome occluded site) as in panel C; Msn1p titrated as in (D).

To further understand the role of ethanol in *FLO11* signaling, we grew the dual-reporter strain in SD ura- media in a range of ethanol concentrations. *FLO11* expression exhibited a graded response to increasing ethanol levels, but the average expression level remained low even at the highest (3%) ethanol concentrations (Figure 6A). The graded response suggested a lack of silencing possibly due to increased Flo8p activity. This led to the hypothesis that at low levels of ethanol (<1%), Flo8p activity eliminates Sfl1p-mediated silencing, but has little effect on expression. Therefore, although *FLO11* expression is OFF (lower right quadrant), the promoter is actually in the competent state. To test this idea, we titrated Class I and II activators in 1% ethanol. Both classes were capable of increasing *FLO11* expression to high levels in a *graded* manner (Figure 6B), implying that silencing no longer occurred in these conditions. The corresponding synthetic activators had a similar effect. In contrast, titration of the synthetic activator mimicking Flo8p resulted in a response similar to ethanol (Figure 6C). Finally, Class I and II activator titrations in a *flo8Δ* in SD ura- with 1% ethanol (Figure 6D) reverted to the behavior seen in SD ura- conditions. Therefore, ethanol controls the extent of silencing at *FLO11* in a Flo8p-dependent manner. Both Flo8p and its synthetic analog affect slow promoter transitions, but neither is a strong conventional activator.

## Discussion

**Figure 7:**
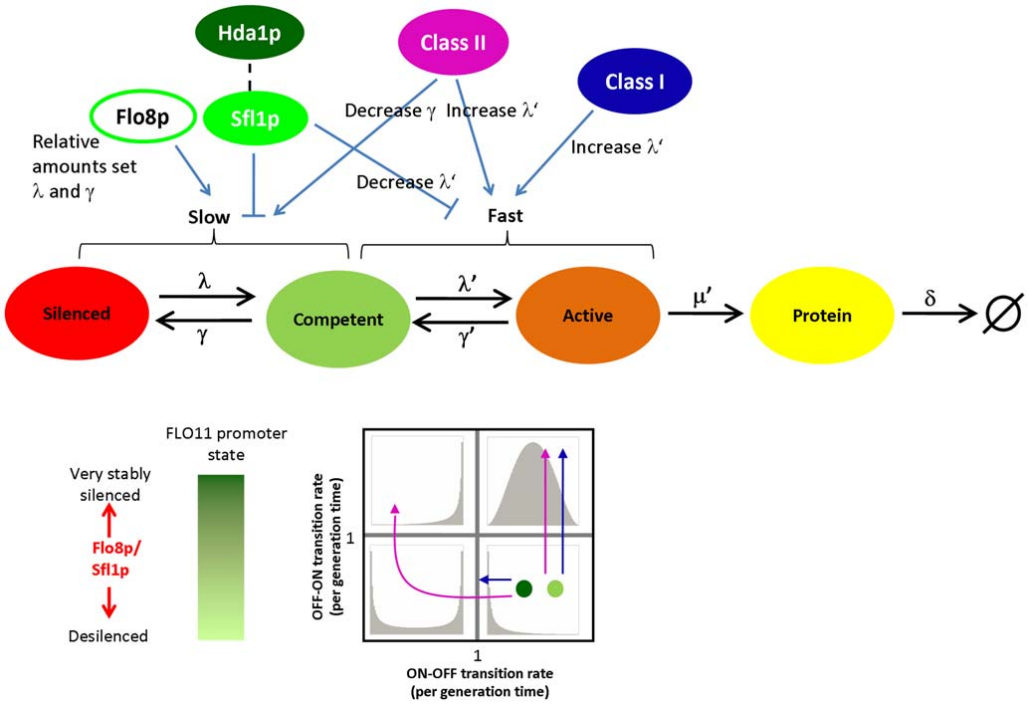
Functional roles of regulators of *FLO11* promoter activation shape the population response. Silencing at the promoter is established by binding of Sfl1p and recruitment of Hda1p. Relative activities of Flo8p and Sfl1p determine the chromatin state of the promoter. The underlying promoter state of an OFF population can be revealed by addition of Class I and II activators (bottom), as Class I activators cannot effectively activate transcription at a silenced promoter, whereas Class II activators can activate expression by sufficiently stabilizing the competent state. At very high levels, Class II activators disrupt silencing; at this point, all cells are also expressing highly. In contrast, an intermediate level of Flo8p activity can “open” the promoter converting the silenced state to a stable competent state, while expression remains low. This opening might be related to chromatin modifications. The combination of Flo8p activation and Class I activators allows the decoupling of chromatin state and expression level, whereas activation by Class II activators alone would not.

The main results of our work are perhaps best understood with reference to the 3-state model in Figure 7, a simple, but useful paradigm for describing the kinetics of epigenetic gene regulation. Regulators can affect either the slow transition rates associated with epigenetic silencing or the fast transition rates associated with conventional gene activation. While a good deal is known about how regulators affect the fast transition rates, less is known about how regulators affect the slow transition rates, and whether effects on slow and fast transition rates are coupled. Our work demonstrates that slow transitions at the two copies of the *FLO11* promoter in diploid yeast occur randomly and independently. Furthermore, we identify the role of various *trans* regulators of *FLO11* in controlling both slow and fast transition rates; it appears that this control is distributed among various “classes” of regulators. Importantly, distributed control enables the cell to shape the diversity of *FLO11* expression within an isogenic population by utilizing different combinations of regulators.

Our demonstration that two copies of the *FLO11* promoter switch slowly and independently builds on previous work demonstrating (1) the *FLO11* promoter is epigenetically regulated [30], and (2) two copies of a partially silenced *URA3* promoter inserted at the mating type loci in *S. cerevisiae* switch independently in a *sir1* background [24]. We now provide evidence that independent switching occurs in a natural gene whose epigenetic regulation is not SIR protein dependent. Given the rich diversity of the *FLO11* gene pool [29] independent switching may be an additional mechanism for generating variation in adhesive phenotype.

To understand how expression heterogeneity at *FLO11* is controlled, we took a functional approach and determined the kinetic role of different regulators on both slow and fast promoter fluctuations. Class I regulates fast promoter fluctuations exclusively. Intrinsic noise measurements confirmed these regulators destabilize the competent state and increase the burst frequency, a common theme for regulators in yeast [19],[20]. Interestingly, we find that the Class II and Flo8p regulate slow promoter fluctuations primarily by stabilizing the competent state. The previous study at the mating type loci found an activator, Ppr1p, could challenge the silenced state and affected both slow transition rates [24], but this was only measured at one level of Ppr1p. It is only by titrating regulators that we were able to clearly discern their functional roles. Whether activators generally affect one or both of the transition rates of silenced genes remains an open question, but likely depends on the mechanism of silencing.

While the regulator titrations indicate that ethanol desilences the promoter via Flo8p, the pathway(s) by which ethanol activates Flo8p is unknown. The simplest mechanism is that long term growth in ethanol activates PKA (specifically Tpk2p), which then activates Flo8p and deactivates Sfl1p. In fact, glucose is not required as the promoter response when Flo8p was titrated in synthetic media with ethanol or glycerol as the sole carbon source was similar to that in SD 1% ethanol (data not shown). However, activation of the PKA pathway via ethanol has not been reported. It is also possible that Flo8p is activated by ethanol via another pathway independent of PKA, although to our knowledge, it is not known to be the target of any other kinase. A third possibility is that ethanol may enable synergistic interactions between Flo8p and other regulators that leads to desilencing, although there is no evidence of Flo8p interacting with any Class I activators. These possibilities could be distinguished by monitoring cyclic AMP levels and the phosphorylation status of Flo8p and Sfl1p in ethanol.

Our results lend support to the idea that binding site position within the *FLO11* promoter can largely determine the kinetic role of the transcriptional regulator. However, the mechanistic description of how binding to particular sites affects slow epigenetic regulation and fast conventional regulation and the molecular nature of the silenced and competent promoter states is still unclear. A mechanistic explanation for the dual roles of Sfl1p is likely the clearest. Binding in the −1200 nucleosome-free region governs epigenetic silencing, possibly by recruiting Hda1p via Tup1p/Ssn6p corepressor [38]. Conventional regulation most likely occurs via Sfl1p binding to the −200 region which contains a putative Sfl1p binding site and has been shown to bind Sfl1p *in vitro*. Indeed, preliminary ChIP experiments suggest Sfl1p is bound to this region *in vivo* (Octavio and Maheshri, unpublished results).

Among the activators, the role of Flo8p and its synthetic analog is perhaps the clearest. Flo8p binding and Sfl1p binding at the −1200 region are likely mutually exclusive because of overlapping binding sites, and so Flo8p can prevent the Sfl1p-mediated establishment of silencing but probably not directly affect conventional Sfl1p repression. This would explain the ability of Flo8p and its corresponding synthetic analog to affect the slow epigenetic transition independently of the conventional activation. In fact, binding of tetR, which lacks the VP16 activation domain of rtTA, to the −1160 tetO site is sufficient to abrograte silencing (data not shown), implying steric hindrance is the major mode of action. Furthermore, any weak activation via Flo8p might be through its known role in binding to the promoters of other Class I activators (including the ones tested here) and presumably upregulating their expression. In this manner, Flo8p activation can put the *FLO11* promoter in a competent, “poised” state whose expression can be controlled by Class I –like regulators. There is evidence, though, that Flo8p can bind to other regions of the promoter [31].

Several possibilities exist for the inability of the Class I activators to challenge the silenced state, yet still regulate the burst frequency of the competent *FLO11* promoter. For example, binding site proximity to the transcriptional start site could play a dominant role. Canonical yeast promoters are typically 150–400 bp with transcription factor binding sites are clustered 100–200 bp from the transcriptional start site [42]; this proximity allows direct interaction with general transcriptional machinery. Therefore, it may be that Class I activators bind in the core region of the *FLO11* promoter, a region that may be inaccessible to transcription factors and/or transcriptional machinery in the silenced state. However, while the Class I synthetic analog binding site is at −350 in a nucleosome occluded region, not all Class I activators have binding sites in this region [31].

A second possibility not mutually exclusive with the first is that Class I activators need not bind in the downstream region but can influence transcription rates via long range (but fast) mechanisms including DNA looping, cryptic transcription, or long range chromatin remodeling. This would provide an explanation for the presence of Class I activator binding sites in these regions that are known to be bound *in vivo* in activating conditions [31]. In addition, it might explain why even at high levels of expression in the absence of silencing the intrinsic noise at the *FLO11* promoter is 10 times higher than that of a similarly highly expressed *PHO84* promoter (data not shown). However, with either explanation, the inability of Class I activators to challenge the silenced state is not clear. Altered chromatin structure or reduced binding site accessibility could be invoked as Class I activator sites tend to be under nucleosomes. However, other than some nucleosome depletion in the core promoter and the −1300 region, no gross nucleosomal rearrangements seem to occur upon silencing (Figure S9) although higher resolution mapping may reveal finer differences.

While we do not know the biochemical intermediates during the slow promoter transitions, the pseudo-first order rates suggest a single slow step, rather than a distributed control mechanism. This is similar to both the partially silenced mating type loci [24] and the epigenetically regulated *agn43* gene in *E. coli* [43]. Possibilities for the slow epigenetic step governing ON to OFF might include Sfl1p binding or Sfl1p-mediated recruitment of silencing factors, among others. Both Class II activators Msn1p and Mss11p are capable of stabilizing the active state, but their localization and activity with respect to the *FLO11* promoter remains unclear [32],[34]. The ability of the Class II synthetic analog to have a similar stabilizing effect on the ON state by binding to the −1470 region as well as the differential acetylation state of that region strongly suggests chromatin remodeling in the upstream region affects accessibility of Class I activators and the transition to the competent state. High resolution mapping of the chromatin state of the entire *FLO11* promoter under various conditions should point toward the biochemical mechanism of the slow promoter transition and will be the focus of future work.

Our findings have implications for the regulation of various subtelomerically encoded gene families known to be epigenetically regulated. This includes the *FLO* gene family [30] and other closely related yeast adhesins [25] such as the *EPA* gene family in the pathogenic yeast *C. glabrata* [44]. Phenotypic variability in *EPA* gene expression might allow *C. glabrata* to rapidly colonize new host tissues and evade immune surveillance. Do such genes turn ON and OFF independently, does it depend on the mechanisms of their silencing (SIR-dependent, etc.), their relative chromosomal locations, or the presence of boundary elements? What promoter transitions do *trans* factors regulate? This understanding will allow the engineering of strains with well-defined levels of phenotypic heterogeneity. Such strains are a prerequisite to quantify what role if any phenotypic heterogeneity has on organismal fitness in natural environments.

## Materials and Methods

### Yeast strains and media

To use the dual-reporter assay to study switching of the *FLO11* promoter, we replaced the *FLO11* ORF in a haploid Σ1278b from the Heitman laboratory [45] with Y*FP-*K*anMX6* or CFP-K*anMX6 *cassettes by PCR integration, and then mated to create diploids. All strains and plasmids used are provided in Table S1 and Table S2.

SD is synthetic defined media with 2% glucose. SD ura- media lacks uracil. SD ura- media with ethanol contains both 2% glucose and a specified amount of ethanol.

### Single cell measurements and analysis

Cells from overnight cultures grown in SD ura- or SD ura- + ethanol were inoculated at an initial OD_600_ between 0.005 and 0.01, and grown for 15–20 hours in the same media. For the titration experiments, these cultures were treated with serial dilutions of doxycyline (0 to 10000 ng/ml) at 30°C in well-agitated deep well 96-well plates. Cells were harvested in mid-late log phase (OD_600_ between 0.5 and 1.5), and placed on ice while other samples were being processed. Expression was measured using a Zeiss AxioObserver microscope with filters optimized for yECitrine, mCherry, and Cerulean (Chroma). Metamorph software (Molecular Devices) was used to analyze images and quantify single cell YFP and CFP fluorescence. Between 500 and 1500 cells were imaged for each sample. Fluorescence levels in the RFP channel was used to discard dead cells (usually <5% of population). Details of data preprocessing and estimation of *λ* and *γ* are given in the Text S1.

In timelapse microscopy experiments, cells were loaded onto the ONIX Microfluidic Platform (CellASIC) with initially ∼10 cells trapped in individual chambers. Media in the ∼10^−5^ µl chambers was constantly replenished at a rate of 10 µl/hr. YFP, CFP, RFP fluorescence and bright field images were obtained every 15 minutes for 20 hours. An example is provided in Video S1. Image stacks were segmented using custom Metamorph journals. Single cell tracking and fluorescence was determined using custom MATLAB routines.

### Chromatin immunoprecipitation assay

Chromatin IP's were done based on the method of [46]. Briefly, ∼40 ml of cells were grown at 30°C in either SD complete or SD leu- to an OD_600_ = 0.8. Lysates from the fixed cells were sonicated to shear the chromatin to an average length of 500 bp, and isolated chromatin was incubated with 2 µl antibodies (Upstate/Millipore) against either histone H3 (Cat. No. 05-928), histone H4 (Cat. No. 05-858), acetylated histone H3 (Cat. No. 07-593) and acetylated histone H4 (Cat. No. 06-866). A sample with no antibodies was also prepared as a control. After reversal of cross-links, DNA from immunoprecipated chromatin was purified and analyzed using quantitative PCR (Applied Biosystems). Primers amplifying the −1.7 to −1.5 kb region of the *FLO11* promoter and primers amplifying a telomeric region in the right arm of chromosome VI [47] as a control for hypoacetylated histone signals were used. Applied Biosystems 7300 software was used to obtain cycle threshold values. All signals from experimental samples were quantified relative to signal from a known amount of genomic DNA from an unmodified, cogenic Σ1287b strain (MLY43, Table S1) that served as a positive control. The ratio of anti-histone/anti-acetylated histone signals was used as a measure of average H3 and H4 acetylation in the region.

### Micrococcal nuclease assay

Micrococcal nuclease assays were performed as in [48]. All cells were grown at 30°C in either SD complete or SD leu- to OD_600_ = 0.5.

## Supporting Information

Figure S1 Nucleosome occupancy data from Figure S9 is plotted along with predicted nucleosome positions from studies predicting nucleosome position genome-wide prediction. The Kaplan et al [17] study provides a computational prediction of nucleosome positioning on any sequence. Only a few nucleosome poor regions bordered by well-defined nucleosomes are predicted, including the −1200 region. The Mavrich et al [18] study used a statistical model to analyze their experimental genome-wide nucleosomal occupancy data. The x-error bars denote how fuzzy the position is. The relative occupancy scale is arbitrary, and absolute number cannot be compared between datasets. However, some general trends in positioned and fuzzy nucleosomes are apparent and this was the basis of Figure 1 in the main text.
(0.46 MB TIF)

Figure S2 *FLO11* expression on solid media - single reporter strains. 10 µL of a mid-log phase culture of various diploid strains with single reporters were spotted on a fresh YPD plate. Labels on the left indicate whether and at which locus the *FLO11* ORF was replaced with a particular fluorescent protein variant. Plates were left at room temperature. Cells from all regions of the spot were sampled (see Text S1) and CFP and YFP expression of these samples was monitored every 2 days by fluorescence microscopy. Density plots for each sample are given, where the x-axis is log CFP fluorescence levels and the y-axis is log YFP fluorescence levels. Cellular autofluorescence can be estimated based on the (first) control strain. Both fluorescence reporters respond equivalently, whether integrated at the A or α locus.
(1.26 MB TIF)

Figure S3 *FLO11* expression on solid media - dual reporter strains. As in panel Figure S2. Four different dual reporter strains were constructed, two with CFP at the α locus and YFP at the A locus, and two in the opposite configuration. Their response is similar, verifying that both reporters are equivalent. Furthermore, the expression distribution of each individual fluorescent reporter is equivalent to the corresponding single reporter strain in Figure S2, verifying independence and the fact that *FLO11* expression doesn't feedback and affect its own expression.
(1.28 MB TIF)

Figure S4 Static snapshots of Y45 cells in YP 1% Ethanol, 2% Glycerol maintained in exponential phase by dilution. The null hypothesis that distributions of YFP fluorescence at each time point are equivalent cannot be rejected (two-way Kolmogorov-Smirnov test, p = 0.60, 0.25, 0.85 for day 1 vs. day 2, day 2 vs. day 3 and day 1 vs. day 3 respectively). Similarly, the null hypothesis that CFP fluorescence distributions at each time point are equivalent cannot be rejected (two-way Kolmogorov-Smirnov test, p = 0.66, 0.36, 0.88 for day 1 vs. day 2, day 2 vs. day 3 and day 1 vs. day 3 respectively).
(0.45 MB TIF)

Figure S5 CFP expression distribution during timelapse. As in Figure 3A, except for CFP rather than YFP.
(0.77 MB TIF)

Figure S6 Switching rates of CFP reporter. As in Figure 3A in the main text, but for CFP rather than YFP. The fit yields switching rates for CFP were *λ/δ* (OFF-ON) = 0.25+0.03 generation^−1^(pink), *γ/δ* (ON-OFF) = 0.90+0.17 generation^−1^ (blue). Error bars correspond to 3 s.d. from the mean calculated by a bootstrap analysis.
(0.64 MB TIF)

Figure S7 OFF(ON)-ON(OFF) switch at *FLO11* is not correlated with cell cycle stage. The time at which a cell born OFF switches ON (A) or the time when a cell born ON switches OFF (B) after its most recent division event occurred is shown for each cell observed to switch during the timelapse experiment in Figure 3A. Points at which the switch occurs do not appear to cluster at any particular position during the cell cycle.
(1.23 MB TIF)

Figure S8 Loss of Hda1p converts the heterogeneous promoter response to activators Mss11p and Phd1p to a graded response. As in Figure 4B and Figure 5E in the main text where Tec1p was titrated in a wildtype (Y45) and *hda1Δ* background respectively, the other activators Phd1p and Mss11p also exhibit a graded response in an *hda1Δ* background (A and B). In the wildtype background, Phd1p (C) like Tec1p is unable to stabilize the ON state enough to enter the bimodal regime, whereas Mss11p (D), like Msn1p (shown in Figure 4B), is able to do so. As in Figure 5F, elimination of silencing in the *hda1Δ* background lowers the threshold level at which Mss11p and Phd1p function (E and F). Error bars represent 3 standard deviations around the mean from bootstrap analysis. Like Tec1p in *hda1Δ* in Figure 5F, both Mss11p and Phd1p control burst frequency (*λ*′) in the absence of silencing, as the square of the intrinsic noise of Phd1p titrated in *hda1Δ* (G) and Mss11p titrated in *hda1Δ* (H) scale with the reciprocal of protein abundance. All titrations were done in SD ura-.
(3.07 MB TIF)

Figure S9 Micrococcal nuclease mapping of *FLO11* was performed on cells grown in conditions where the promoter was completely silenced (growth of wildtype Y45 in SD complete) in (A) or completely active (growth of an *sfl1Δ* strain in SD complete plus 2% glucose) in (B). The overall structure agrees well with the in silico predictions in Figure 1. Although nucleosomal occupancy in the −1300 bp region and the −150 bp region appears to be further depleted in the active state, there is surprisingly no gross rearrangement of nucleosomal structure between the silenced and active state. Error bars are standard error from triplicate quantitative PCR samples.
(0.55 MB TIF)

Table S1 Yeast strains used in study.
(0.05 MB DOC)

Table S2 Plasmids used in study.
(0.03 MB DOC)

Text S1 Supplemental discussion.
(0.11 MB DOC)

Video S1 Movie of cell growth within the microfluidic device. Red and green represent CFP and YFP expression respectively. Both ON to OFF and OFF to ON transitions for each copy of the *FLO11* promoter are readily seen in this video. Each frame represents 15 minutes elapsed and the total movie represents 20 hours of growth.
(3.83 MB AVI)
