# Supplementary figures and images for: Epigenetic and Conventional Regulation Is Distributed among Activators of FLO11 Allowing Tuning of Population-Level Heterogeneity in Its Expression
Source: PLoS Genet. 2009 Oct 2;5(10):e1000673. doi: 10.1371/journal.pgen.1000673 (PMC2745563; doi:10.1371/journal.pgen.1000673)

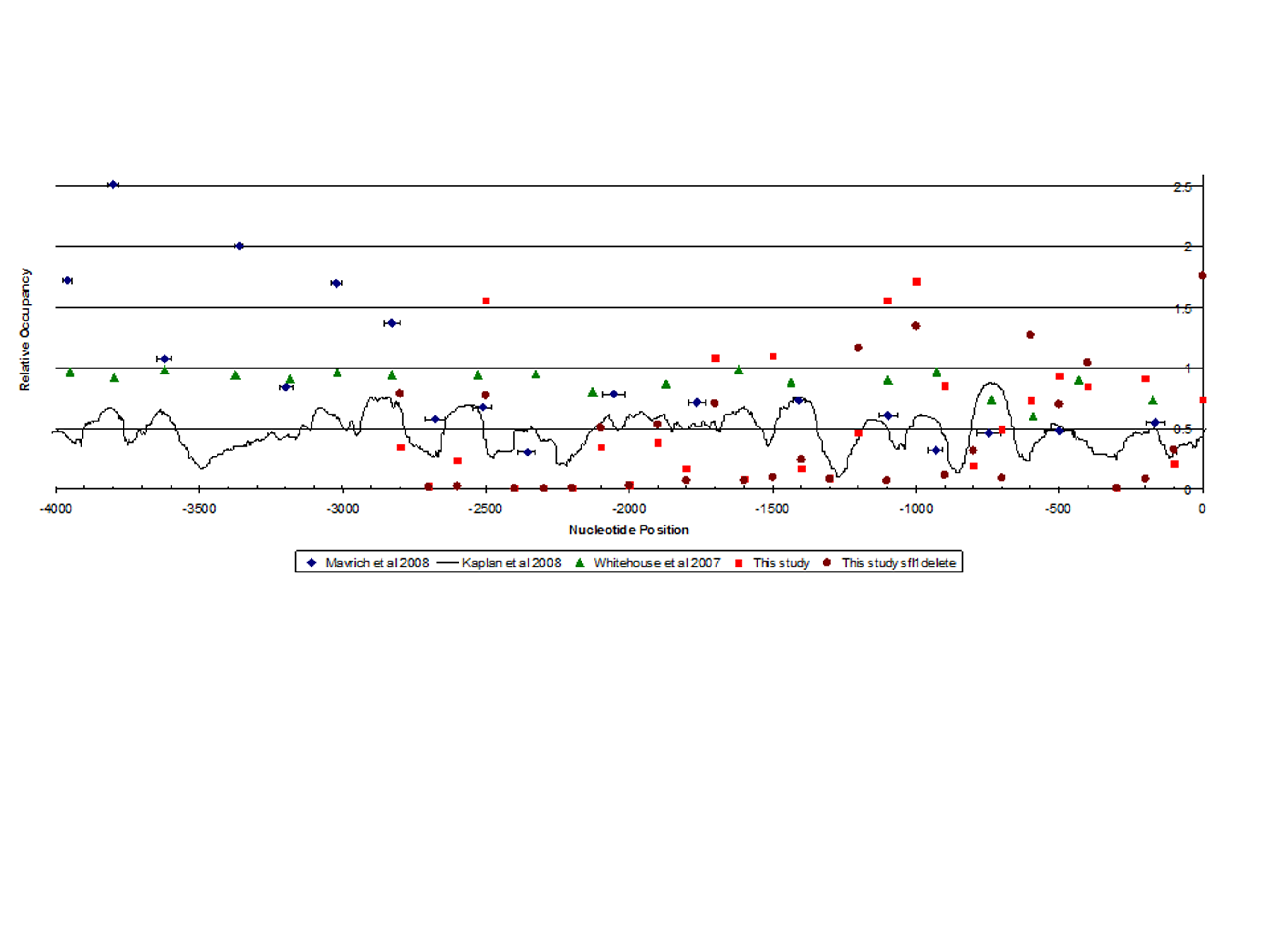

Supplement: Figure S1 — Nucleosome occupancy data from Figure S9 is plotted along with predicted nucleosome positions from studies predicting nucleosome position genome-wide prediction. The Kaplan et al [17] study provides a computational prediction of nucleosome positioning on any sequence. Only a few nucleosome poor regions bordered by well-defined nucleosomes are predicted, including the −1200 region. The Mavrich et al [18] study used a statistical model to analyze their experimental genome-wide nucleosomal occupancy data. The x-error bars denote how fuzzy the position is. The relative occupancy scale is arbitrary, and absolute number cannot be compared between datasets. However, some general trends in positioned and fuzzy nucleosomes are apparent and this was the basis of Figure 1 in the main text. (0.46 MB TIF) [file pgen.1000673.s001.tif]

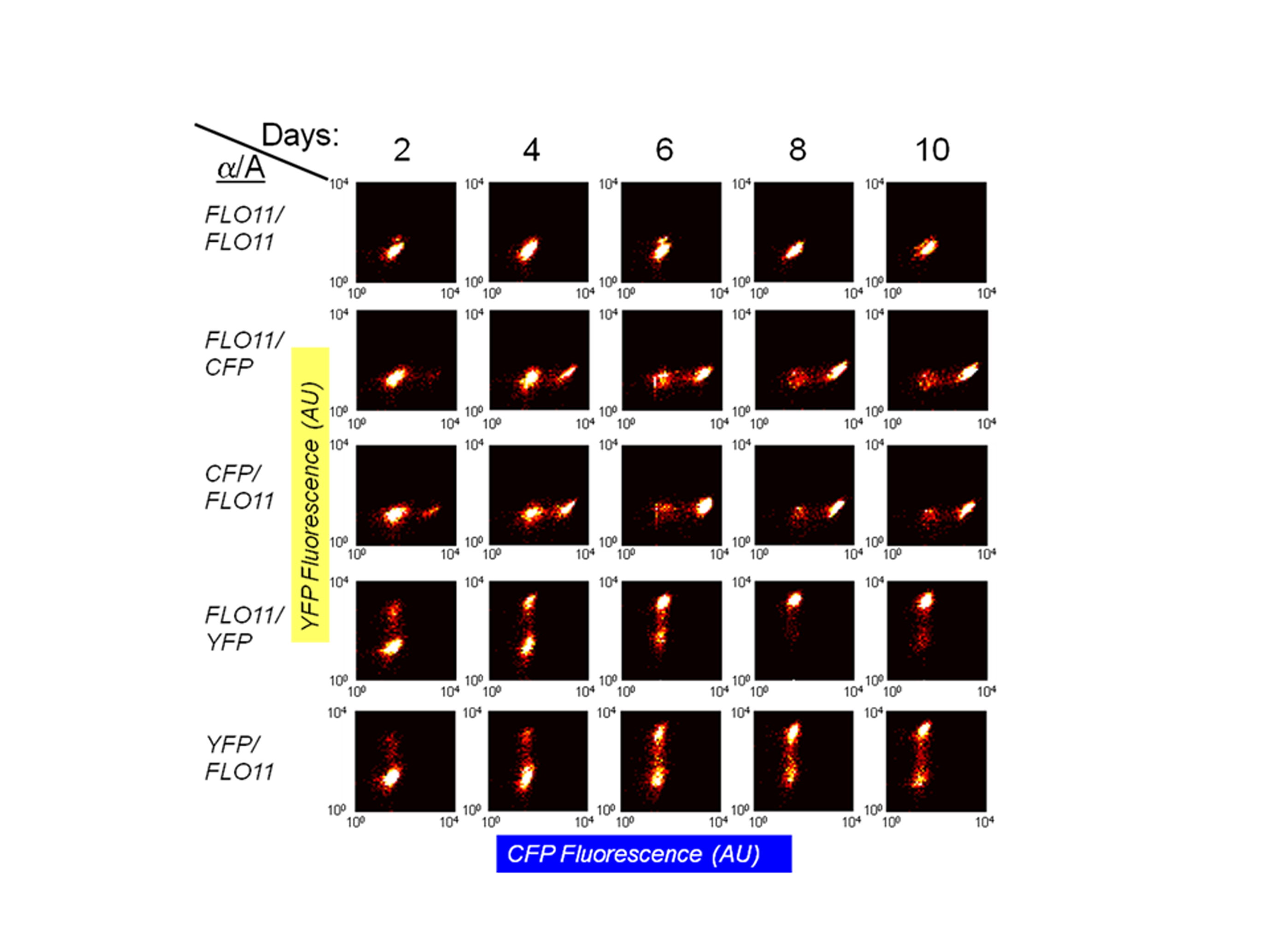

Supplement: Figure S2 — FLO11 expression on solid media - single reporter strains. 10 µL of a mid-log phase culture of various diploid strains with single reporters were spotted on a fresh YPD plate. Labels on the left indicate whether and at which locus the FLO11 ORF was replaced with a particular fluorescent protein variant. Plates were left at room temperature. Cells from all regions of the spot were sampled (see Text S1) and CFP and YFP expression of these samples was monitored every 2 days by fluorescence microscopy. Density plots for each sample are given, where the x-axis is log CFP fluorescence levels and the y-axis is log YFP fluorescence levels. Cellular autofluorescence can be estimated based on the (first) control strain. Both fluorescence reporters respond equivalently, whether integrated at the A or α locus. (1.26 MB TIF) [file pgen.1000673.s002.tif]

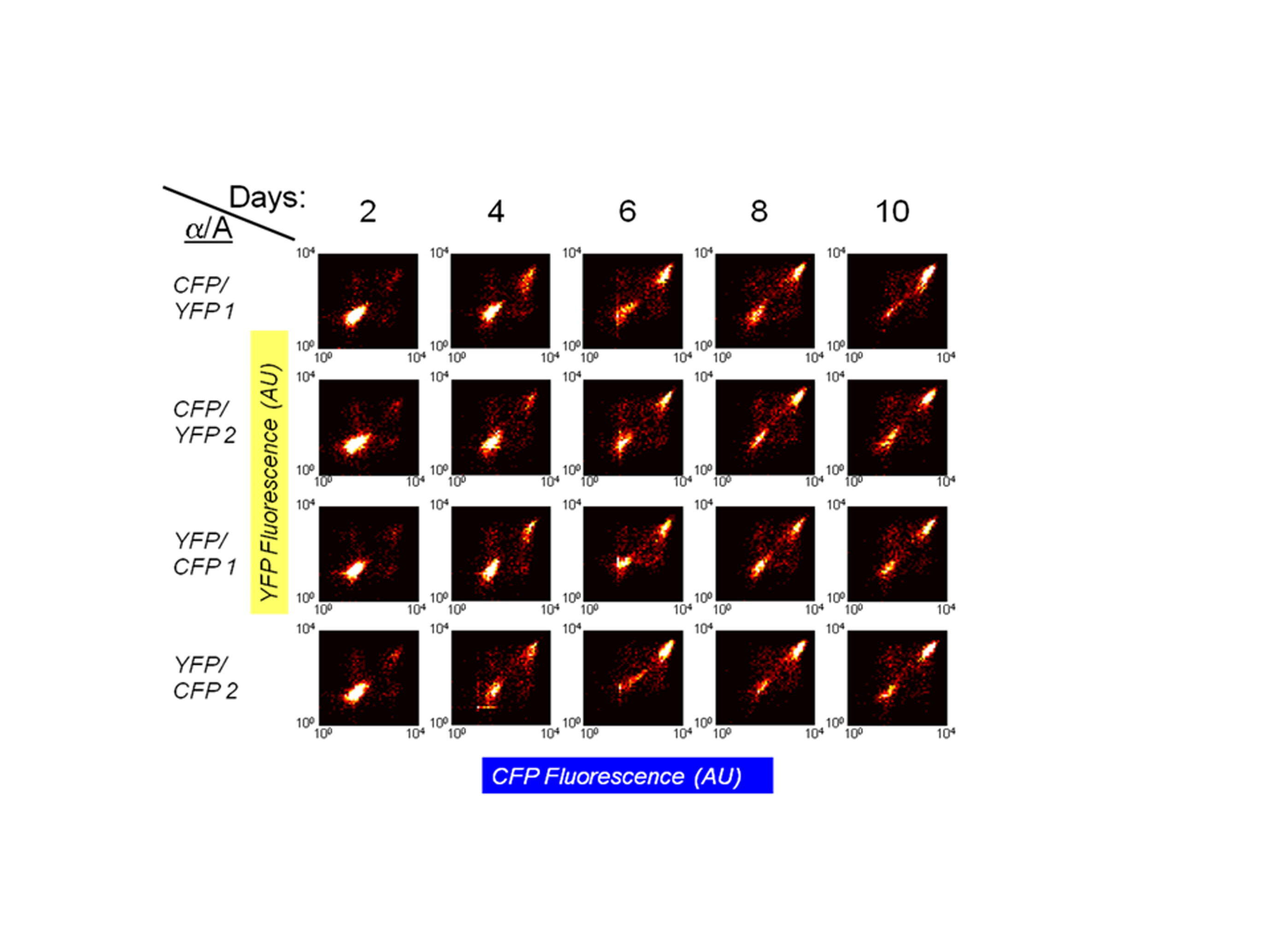

Supplement: Figure S3 — FLO11 expression on solid media - dual reporter strains. As in panel Figure S2. Four different dual reporter strains were constructed, two with CFP at the α locus and YFP at the A locus, and two in the opposite configuration. Their response is similar, verifying that both reporters are equivalent. Furthermore, the expression distribution of each individual fluorescent reporter is equivalent to the corresponding single reporter strain in Figure S2, verifying independence and the fact that FLO11 expression doesn't feedback and affect its own expression. (1.28 MB TIF) [file pgen.1000673.s003.tif]

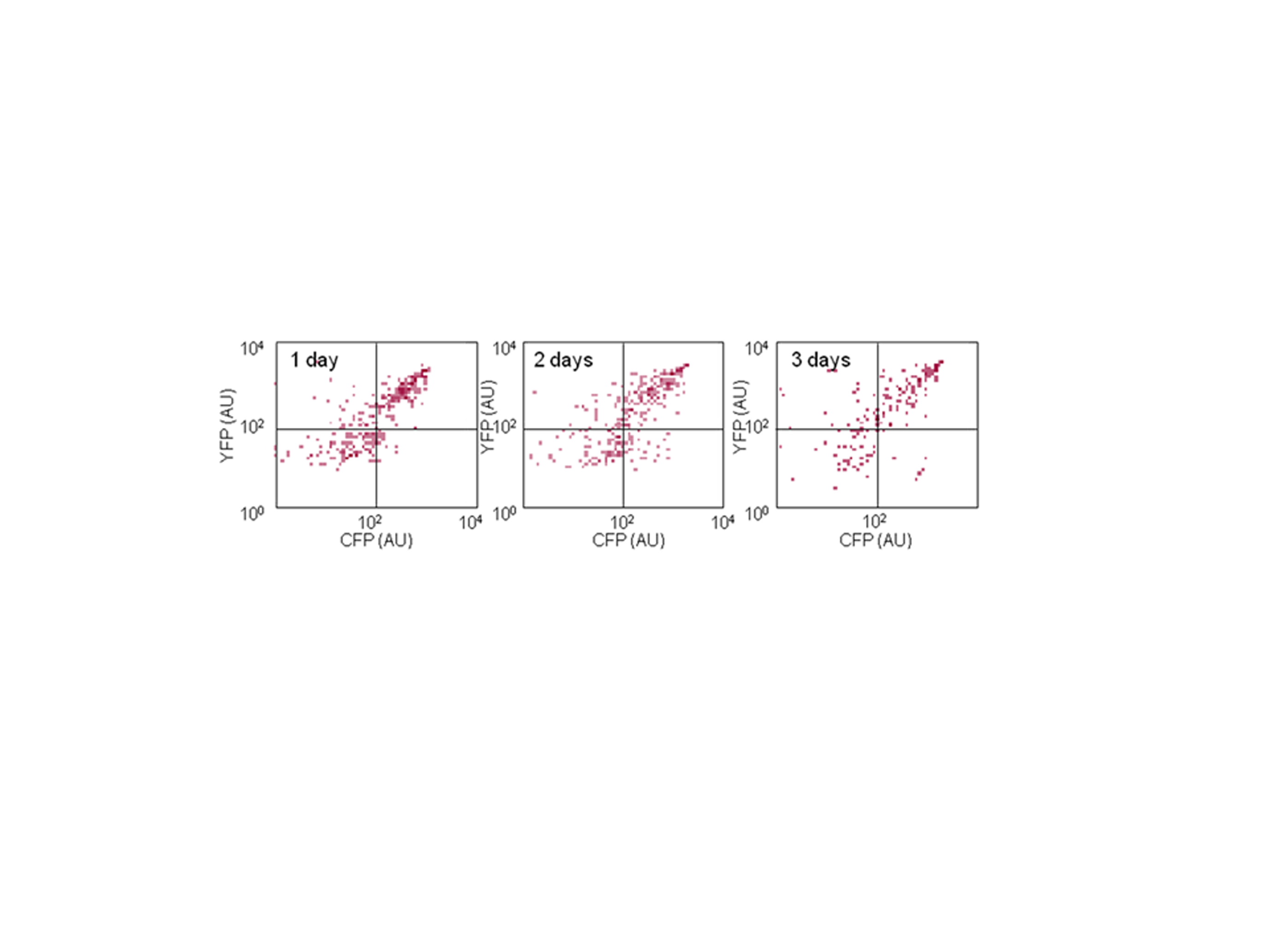

Supplement: Figure S4 — Static snapshots of Y45 cells in YP 1% Ethanol, 2% Glycerol maintained in exponential phase by dilution. The null hypothesis that distributions of YFP fluorescence at each time point are equivalent cannot be rejected (two-way Kolmogorov-Smirnov test, p = 0.60, 0.25, 0.85 for day 1 vs. day 2, day 2 vs. day 3 and day 1 vs. day 3 respectively). Similarly, the null hypothesis that CFP fluorescence distributions at each time point are equivalent cannot be rejected (two-way Kolmogorov-Smirnov test, p = 0.66, 0.36, 0.88 for day 1 vs. day 2, day 2 vs. day 3 and day 1 vs. day 3 respectively). (0.45 MB TIF) [file pgen.1000673.s004.tif]

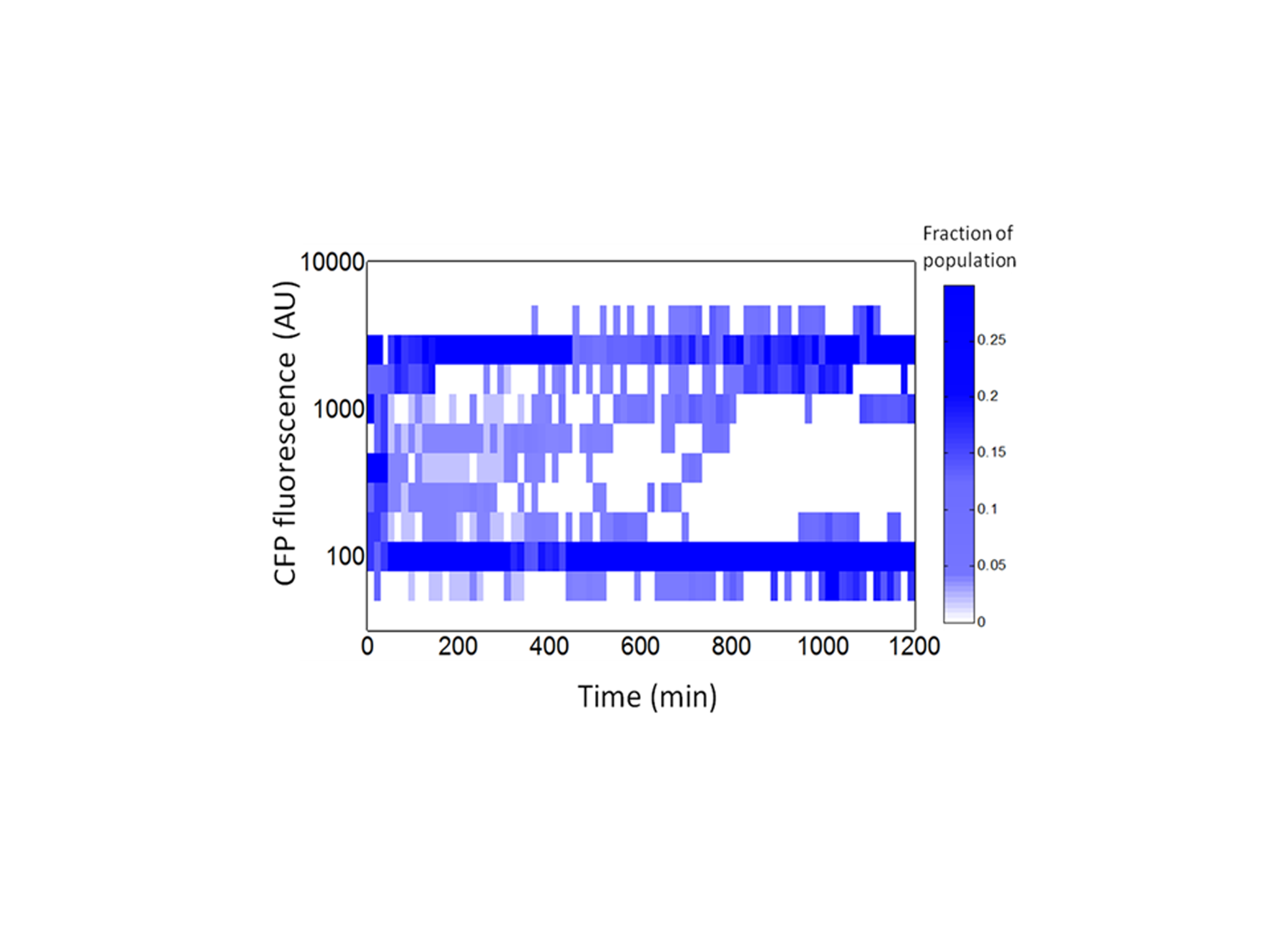

Supplement: Figure S5 — CFP expression distribution during timelapse. As in Figure 3A, except for CFP rather than YFP. (0.77 MB TIF) [file pgen.1000673.s005.tif]

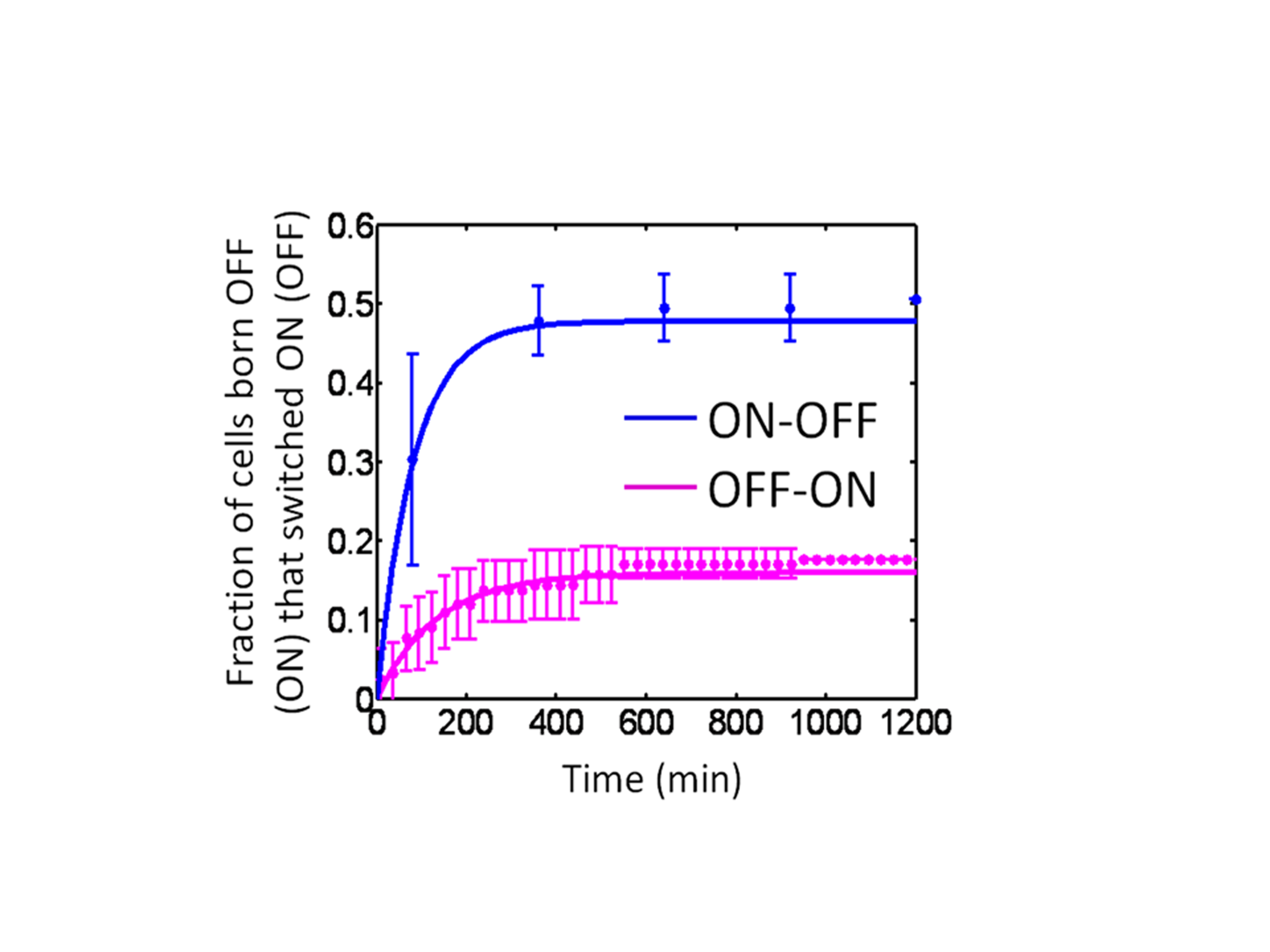

Supplement: Figure S6 — Switching rates of CFP reporter. As in Figure 3A in the main text, but for CFP rather than YFP. The fit yields switching rates for CFP were λ/δ (OFF-ON) = 0.25+0.03 generation−1(pink), γ/δ (ON-OFF) = 0.90+0.17 generation−1 (blue). Error bars correspond to 3 s.d. from the mean calculated by a bootstrap analysis. (0.64 MB TIF) [file pgen.1000673.s006.tif]

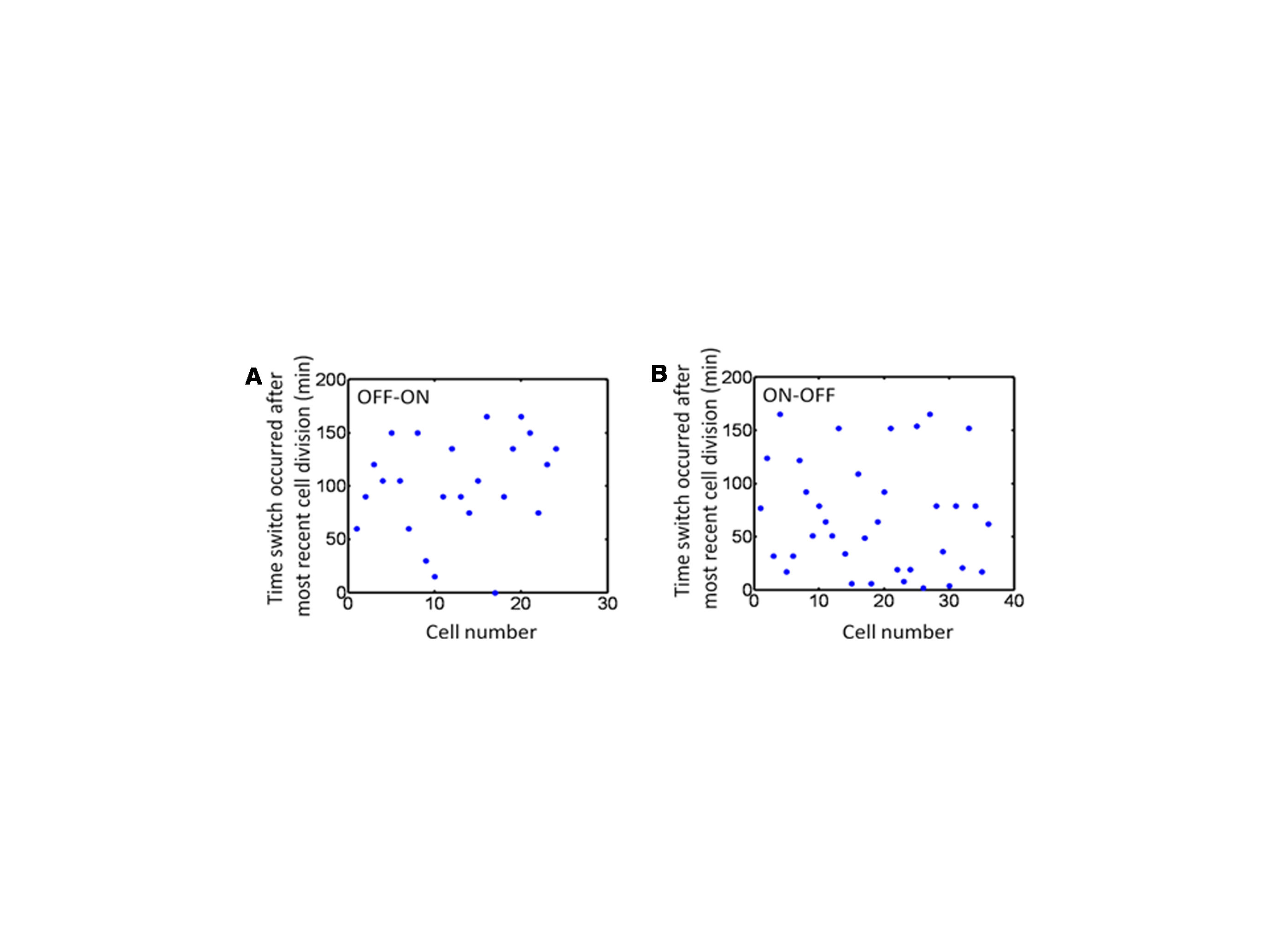

Supplement: Figure S7 — OFF(ON)-ON(OFF) switch at FLO11 is not correlated with cell cycle stage. The time at which a cell born OFF switches ON (A) or the time when a cell born ON switches OFF (B) after its most recent division event occurred is shown for each cell observed to switch during the timelapse experiment in Figure 3A. Points at which the switch occurs do not appear to cluster at any particular position during the cell cycle. (1.23 MB TIF) [file pgen.1000673.s007.tif]

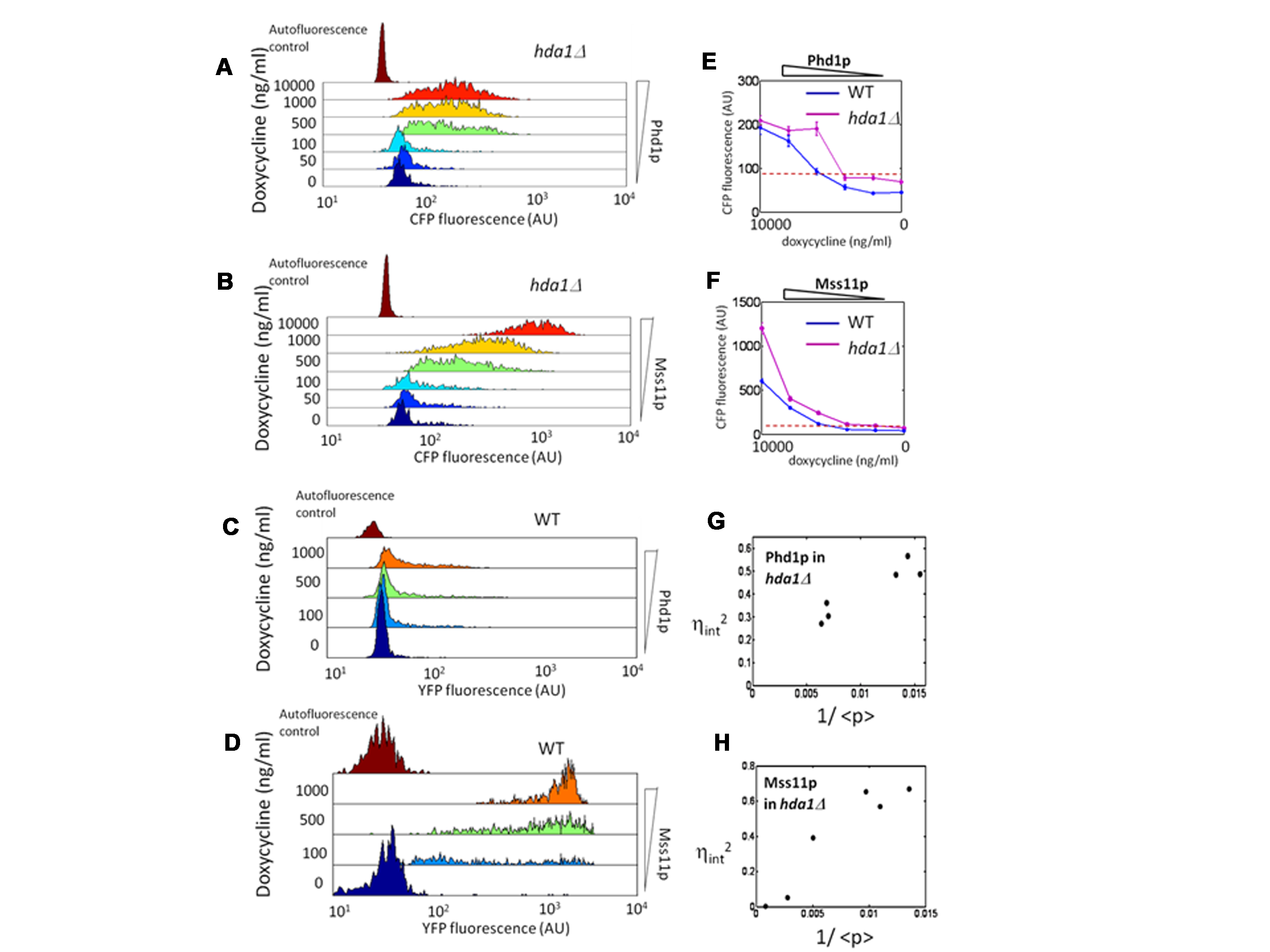

Supplement: Figure S8 — Loss of Hda1p converts the heterogeneous promoter response to activators Mss11p and Phd1p to a graded response. As in Figure 4B and Figure 5E in the main text where Tec1p was titrated in a wildtype (Y45) and hda1Δ background respectively, the other activators Phd1p and Mss11p also exhibit a graded response in an hda1Δ background (A and B). In the wildtype background, Phd1p (C) like Tec1p is unable to stabilize the ON state enough to enter the bimodal regime, whereas Mss11p (D), like Msn1p (shown in Figure 4B), is able to do so. As in Figure 5F, elimination of silencing in the hda1Δ background lowers the threshold level at which Mss11p and Phd1p function (E and F). Error bars represent 3 standard deviations around the mean from bootstrap analysis. Like Tec1p in hda1Δ in Figure 5F, both Mss11p and Phd1p control burst frequency (λ′) in the absence of silencing, as the square of the intrinsic noise of Phd1p titrated in hda1Δ (G) and Mss11p titrated in hda1Δ (H) scale with the reciprocal of protein abundance. All titrations were done in SD ura-. (3.07 MB TIF) [file pgen.1000673.s008.tif]

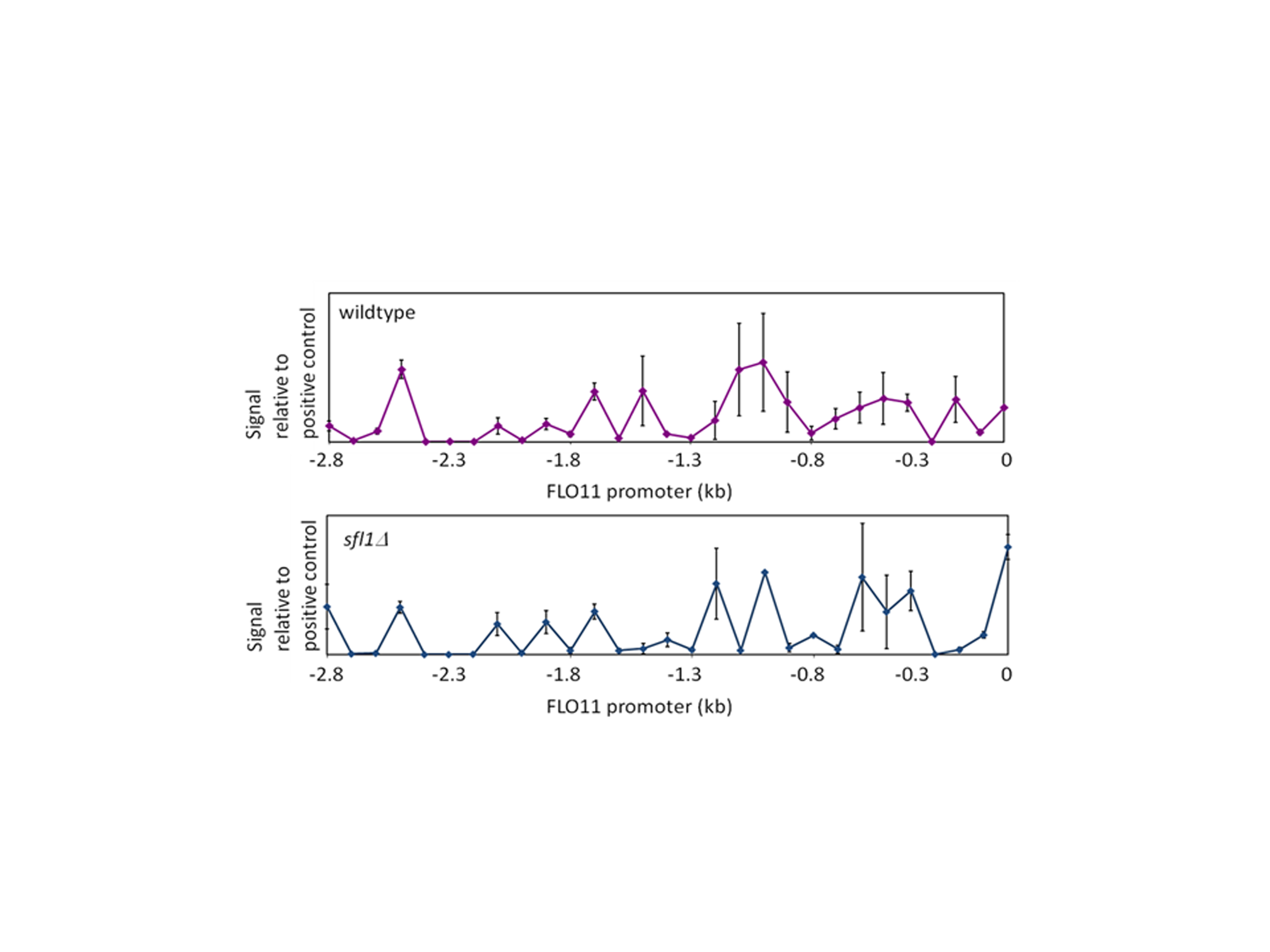

Supplement: Figure S9 — Micrococcal nuclease mapping of FLO11 was performed on cells grown in conditions where the promoter was completely silenced (growth of wildtype Y45 in SD complete) in (A) or completely active (growth of an sfl1Δ strain in SD complete plus 2% glucose) in (B). The overall structure agrees well with the in silico predictions in Figure 1. Although nucleosomal occupancy in the −1300 bp region and the −150 bp region appears to be further depleted in the active state, there is surprisingly no gross rearrangement of nucleosomal structure between the silenced and active state. Error bars are standard error from triplicate quantitative PCR samples. (0.55 MB TIF) [file pgen.1000673.s009.tif]
