# Supplementary material for: Epigenetic and Conventional Regulation Is Distributed among Activators of FLO11 Allowing Tuning of Population-Level Heterogeneity in Its Expression
Source: PLoS Genet. 2009 Oct 2;5(10):e1000673. doi: 10.1371/journal.pgen.1000673 (PMC2745563; doi:10.1371/journal.pgen.1000673)
Supplement: Table S1 — Yeast strains used in study. (0.05 MB DOC) [file pgen.1000673.s010.doc]

**SUPPLEMENTAL TABLE 1: Yeast strains used in study**

**Strains constructed from Σ1278 ML *ura3-52*, *leu2Δ*::*hisG* ^[1]^**

| Strain | ***Genotype* (Source)** |
| --- | --- |
| MLY42 | **Σ1278b** *MAT* α *ura3-52 leu2Δ::hisG* ^[1]^ |
| MLY43 | **Σ1278b** *MAT* **a** *ura3-52 leu2Δ::hisG* |
| Y35 | *MAT* α *flo11Δ*::CFP-KanMX6 *ura3-52* *leu2Δ*::*hisG* (GAL HO switched Y37)** |
| Y36 | *MAT* α *flo11Δ*::YFP-KanMX6 *ura3-52* *leu2Δ*::*hisG* (GAL HO switched Y38)** |
| Y37 | *MAT* a *flo11Δ*::CFP-KanMX6 *ura3-52* *leu2Δ*::*hisG* |
| Y38 | *MAT* a *flo11Δ*::YFP-KanMX6 *ura3-52* *leu2Δ*::*hisG* |
| Y39 | *MAT* a/α  *ura3-52/ura3-52 leu2Δ::hisG/leu2Δ::hisG* |
| Y40 | *MAT* a/α *FLO11/flo11Δ::CFP-KanMX6 ura3-52/ura3-52 leu2Δ::hisG/leu2Δ::hisG* |
| Y41 | *MAT* a/α *flo11Δ::CFP-KanMX6/FLO11 ura3-52/ura3-52 leu2Δ::hisG/leu2Δ::hisG* |
| Y42 | *MAT* a/α *FLO11/flo11Δ::YFP-KanMX6 ura3-52/ura3-52 leu2Δ::hisG/leu2Δ::hisG* |
| Y43 | *MAT* a/α *flo11Δ::YFP-KanMX6/FLO11 ura3-52/ura3-52 leu2Δ::hisG/leu2Δ::hisG* |
| Y44 | *MAT* a/α *flo11Δ::YFP-KanMX6/flo11Δ::CFP-KanMX6 ura3-52/ura3-52 leu2Δ::hisG/leu2Δ::hisG* |
| Y45 | *MAT* α/**a** *flo11Δ::YFP-KanMX6*/*flo11Δ::CFP-KanMX6 ura3-52/ura3-52 leu2::hisG leu2Δ::hisG* |
| Y92 | *MAT* α/a  *flo11Δ::YFP-KanMX6/flo11Δ::CFP-KanMX6 flo8Δ::LEU2/flo8Δ::LEU2 ura3-52/ura3-52 leu2 Δ::hisG/leu2Δ::hisG* |
| Y93 | *MAT* α/a *flo11Δ::YFP-KanMX6/ flo11Δ::CFP-KanMX6 sfl1Δ::LEU2/sfl1 Δ::LEU2 ura3-52/ura3-52 leu2Δ::hisG/leu2Δ::hisG* |
| Y171 | *MAT* α/**a** *flo11Δ::YFP-KanMX6 / flo11Δ::CFP-KanMX6 hda1Δ::LEU2/hda1Δ::LEU2 ura3-52/ura3-52 leu2Δ::hisG/leu2Δ::hisG* |
| Y197 | *MAT* α/a *flo11Δ::YFP-KanMX6 / flo11Δ::CFP-KanMX6 mss11Δ::LEU2 /mss11Δ::LEU2 ura3-52/ura3-52 leu2 Δ::hisG/ leu2Δ::hisG* |
| Y253 | *MAT* α/a *flo11Δ::YFP-KanMX6 / flo11Δ::CFP-KanMX6 -350 region of FLO11 promoter::tetO site/-350 region of FLO11 promoter::tetO site ura3-52/ura3-52 leu2 Δ::hisG/ leu2 Δ::hisG* |
| Y254 | *MAT* α/a *flo11Δ::YFP-KanMX6 / flo11Δ::CFP-KanMX6 -1400 region of FLO11 promoter::tetO site/-1400 region of FLO11 promoter::tetO site ura3-52/ura3-52 leu2 Δ::hisG/ leu2 Δ::hisG* |
| **Y255** | *MAT* α/a  *flo11Δ::YFP-KanMX6 / flo11Δ::CFP-KanMX6 -1470 region of FLO11 promoter::tetO site/-1470 region of FLO11 promoter::tetO site ura3-52/ura3-52 leu2 Δ::hisG/ leu2 Δ::hisG* |

*** All strains constructed are in the Σ1278b background and cogenic to MLY43.**

**** Haploid strains Y35, Y36 were constructed by HO-mediated mating type switching of strains Y37, Y38, to ensure all diploids were created from isogenic haploid strains.**

**NOTE:** Strains with selected *FLO11* regulators knocked out were created by PCR integration to delete the regulator ORF in the YFP and CFP haploids and then mated to obtain diploids. Yeast were transformed by the PEG/Lithium Acetate method ^[2]^ and all integrations were verified through colony PCR.
