# Supplementary material for: Epigenetic and Conventional Regulation Is Distributed among Activators of FLO11 Allowing Tuning of Population-Level Heterogeneity in Its Expression
Source: PLoS Genet. 2009 Oct 2;5(10):e1000673. doi: 10.1371/journal.pgen.1000673 (PMC2745563; doi:10.1371/journal.pgen.1000673)
Supplement: Table S2 — Plasmids used in study. (0.03 MB DOC) [file pgen.1000673.s011.doc]

**SUPPLEMENTAL TABLE 2: Plasmids used in study**

*Plasmid Description*

pYC *CEN URA3* ADH1 promoter-rtTA

pYCFlo8 7xtetO site (XhoI/BamHI), *FLO8 ORF* (BamHI/NotI) in pYC

pYCSfl1 7xtetO site (XhoI/BamHI), *SFL1 ORF* (BamHI/NotI) in pYC

pYCPhd1 7xtetO site (XhoI/BamHI), *PHD1 ORF* (BamHI/NotI) in pYC

pYCTec1 7xtetO site (XhoI/BamHI), *TEC1 ORF* (BamHI/NotI) in pYC

pYCSte12 7xtetO site (XhoI/BamHI), *STE12 ORF* (BamHI/NotI) in pYC

pYCMsn1 7xtetO site (XhoI/BamHI), *MSN1 ORF* (BamHI/NotI) in pYC

pYCMss11 7xtetO site (XhoI/BamHI), *MSS11 ORF* (BamHI/NotI) in pYC
