# Supplementary material for: Epigenetic and Conventional Regulation Is Distributed among Activators of FLO11 Allowing Tuning of Population-Level Heterogeneity in Its Expression
Source: PLoS Genet. 2009 Oct 2;5(10):e1000673. doi: 10.1371/journal.pgen.1000673 (PMC2745563; doi:10.1371/journal.pgen.1000673)
Supplement: Text S1 — Supplemental discussion. (0.11 MB DOC) [file pgen.1000673.s012.doc]

**SUPPLEMENTAL DISCUSSION**

***FLO11* signaling on solid media**

We originally encountered the mixed expression states when analyzing colonies of the dual-reporter strain (Y45) grown on rich solid media (YPD, yeast extract, peptone, dextrose) for several days. Since colony growth on a complex solid media may be a more physiologically relevant condition for the adhesion and filamentation response, we monitored reporter gene expression from the *FLO11* locus over the course of 10 days. To do so, we took an exponentially growing culture of the dual-reporter strain and various other control strains and spotted 10 μL containing 2 x 10^3^ cells in 3 separate locations on YPD plates. These plates were left at room temperature for 10 days. Every two days, a mixture of cells originating from each spot was analyzed. In an effort to sample the “average” response of the colony rather than that of individual regions in the colonies, cells were collected by lightly tracing an X pattern in the spot using a toothpick. This insured that cells from both the periphery and edges of the spot were analyzed. We did this experiments with biological duplicates (different patches of the same strain) to verify that this technique yielded reproducible results (Figures S2, S3).

***FLO11* expression is not dependent on Flo11p and alleles are independent and equivalent.**

We created several different control strains to insure that 1) each fluorescent allele was equivalent, 2) each fluorescent allele was equivalent regardless of the haploid mating type from which it originated 3) each fluorescent allele’s expression did not depend on the presence or absence of the other allele, 4) each fluorescent allele’s expression was not affected by the presence of *FLO11*. For 1), compare Figure S2, row 2,3 with 4,5 Figure S3, any row. At 10 days, CFP and YFP levels are not quite equivalent on solid media, which we attribute to difficulties in detection and/or stability. This is never the case in liquid culture (for example, see Figure 2). For 2) compare Figure S2 row 2 with 3 or row 4 with 5. The variability in response between each pair is consistent with variability we see in biological duplicates (see Figure S3). For 3) and 4) compare Figure S2 rows 2,3 or 4,5 versus the marginal CFP or YFP expression in the diploids (Figure S3). The proportion of cells that turn ON for either YFP of CFP is similar with or without *FLO11*.
***FLO11* expression in various environmental conditions**

For the analysis in the main text of the paper, we were careful to always grow cells in SD media and harvest them in mid-log phase. This was to facilitate comparisons between samples and ensure that we were in a steady-state condition. We also analyzed expression from the dual-reporter strain in the following additional media conditions: YP + 2% glucose, 3% glycerol, 3% ethanol, 6% ethanol, YP 2% galactose, 2% raffinose; SLAD (synthetic low ammonium dextrose (2%)); SLARG (synthetic low ammonium galactose (2%)/raffinose(2%)); SD (2% glucose); YPD (2% glucose), SD (2% glucose), SLAD, SLARG + 100 μM tryptophol + 100 μM phenylethanol. The last two sets were performed due to a recent report that certain aromatic alcohols could influence *FLO11* expression [3]. All rich media (YP) conditions yielded a heterogeneous response that persisted as the culture approached saturation (data available upon request). However, in YPD *FLO11* became active near diauxic shift, presumably as glucose levels decreased. For SLAD and SLARG conditions, we saw very little *FLO11* expression. Because the low nitrogen condition in these two media are known to lead to filamentation [4] on solid media, we also grew the dual-reporter strain on SLAD and SLARG plates, and monitored expression in the colony every two days for two weeks. We never observed significant *FLO11* expression (data not shown) suggesting that *FLO11* remained silenced. We are not certain why this was the case. Finally, the aromatic alcohols had very little if any effect on expression.

**Fluorescence time trace analysis**

For the timelapse experiment, cells (Y45) were grown in YP 1% ethanol 2% glycerol. This condition was selected because previous static snapshots revealed that approximately half the population was ON, so observing sufficient numbers of OFF and ON cells in the experiment would be easier. Prior to transfer to the microfluidic chamber, cells had been growing exponentially for >12 hrs. Upon transfer, the time evolution of the YFP expression is given in Figure 3A. At early times, only small numbers of cells are monitored, but within 10 hrs enough cells are present such that the steady-state expression distribution is indistinguishable to the pre-transfer distribution (two-sample KS test, p = 0.19 for CFP, p = 0.21 for YFP). We focused on microcolonies at the inlet only to ensure that the microenvironment was constant and uniform. Data was collected from three experiments, each run on a separate day.

Custom scripts in Metamorph were used to segment images to identify cells and generate masks. Fluorescence images from the experiment and the cell masks were loaded into MATLAB (Mathworks). Custom MATLAB routines were used to further process the masks and track individual cells. Processed tracks were manually reviewed and corrected if necessary (typically in frames with high cell density).

The observation length of each cell within a growing population of cells is not identical, and this observational bias *must* be considered for an accurate estimate of the switching rates. To determine waiting times for cells to switch from OFF to ON, we used a method similar to Kaufmann et al [5]. We took the time between the birth of a cell that was OFF and the time fluorescence signal sharply increases, and accounted for the 20 minute protein maturation for both CFP and YFP, which mature at similar rates [5]. Because the sudden increase in fluorescence signal from the production of our reporter proteins was easily distinguishable from background autofluorescence, we could clearly determine the moment a cell switches ON, within the 15 minute time resolution of our experiment. We therefore fit the distribution of OFF-ON waiting times using a bin size of 15 minutes.

We did not have as good a resolution for determining the time that a cell switches OFF. Because of the long-lived nature of our reporters, even after a cell switches OFF we only observe a sharp decrease in fluorescence signal when the cell divides. Prior to the division event, it is difficult to determine whether small fluctuations in the fluorescence are due to real changes in the protein production or other sources of noise such as photobleaching and tiny drifts in the z-direction during image capture. These fluctuations are negligible compared to the fluorescence change upon division. Therefore, we estimated the time a cell switched OFF by fitting the exponential decay of the fluorescence signal due to dilution from growth and extrapolating to determine the initial point in time when the signal began to decay. Because of the higher uncertainty in the exact moment the cell switched off in the period between division events, we fit the distribution of ON-OFF waiting times using a bin size of 158 minutes (the mean doubling time of the cells in this growth condition).

**Calculation of switching rates from time traces**

If the switching of the *FLO11* promoter is a Poisson process, the distribution of waiting times *t* between ON (OFF) and OFF (ON) states will be exponential, where λ is the switching rate (or, 1/λ is the mean waiting time between switching):

$f(t)=\lambda e^{-\lambda t}$ (exponential PDF) (1)

Let F(*t*) = probability that switch occurs before some time *t*. Then:

$F(t)=1-e^{-\lambda t}$(exponential CDF) (2)

Fitting the waiting times between ON (OFF) and OFF (ON) states to the above distribution would be valid only if we could observe every cell for an infinitely long time. However, we only observe cells for a finite time; moreover, in the limit of infinitely many cells, the observation time intervals are exponentially distributed, as cells are continuously born during the experiment.

To account for the uneven observation time intervals, we assume that the time intervals are exponentially distributed as follows, an assumption that was reasonably accurate for the number of cells in our experiment:

$F(T)=\delta e^{-\delta T}$ (3)

Where T = observation time, δ = mean growth rate of cells

The probability that a cell switches in some time *t*’ is then sum of equations (4) and (5):

1. the probability that the switch occurs within *t*’ and that we observed the cell for *t*’, which is given by:

$\int_{o}^{t^{'}} (1-e^{-\lambda t})\delta e^{-\delta t}dt$ (4)

1. the probability that the switch occurs within *t*’ and we observed the cell longer than *t*’, which is:

$(1-e^{-\lambda t^{'}})e^{-\delta t^{'}}$ (5)

The total probability is then:

P (cell switches by time *t*) = $\frac{\lambda}{\lambda+\delta}e^{-\left(\lambda+\delta\right)t}$ (6)

This is not a true cumulative distribution function as it will never go to 1 at long times, since cells are always being born and the observation time intervals of the newly born cells are short.

We fit the above probability density to the waiting times obtained from timelapse data using nonlinear least square optimization (fminsearch) in custom MATLAB routines.

**Fitting static fluorescence distribution to a stochastic kinetic model**

The solution to the stochastic kinetic model [6] is a Beta distribution, which can assume one of the four distinct shapes shown in Figure 4A, depending on the values of the parameters λ and γ (OFF-ON and ON-OFF transition rates). The model distribution accounts for variation in expression that arises solely from random promoter fluctuations between the ON and OFF states. In our experiments, additional factors such as cell-to-cell variation in the production rate μ between individual cells, intrinsic noise in mRNA transcription and translation, autofluorescence, and experimental error from the imaging equipment also contribute to the distribution we obtain, creating “noisy” ON and OFF peaks.

Intrinsic noise in mRNA production could be incorporated by convolving a Poisson distribution for mRNA levels with the Beta distribution. However, because of the separation in timescales, variation in protein levels from fast mRNA fluctuations is small compared to variation from the silenced promoter slowly switching between OFF and ON states. Similarly, extrinsic variation in production rates between different cells is smaller than the difference between ON and OFF cells, as indicated by our ability to observe “side populations”. Error from the camera and imaging equipment are negligible; autofluorescence can be accounted for by kernel density estimation. Because they are small, these extra sources of variation present in our raw experimental distribution do not change its qualitative shape; in almost all cases, we can easily distinguish bimodal distributions from unimodal ON or unimodal OFF distributions.

The noisy ON and OFF peaks, however, can complicate fitting of the raw distribution data to the model. Prior to fitting, we first applied a kernel density estimate to the raw data, where the kernel is the autofluorescence distribution. We observed the YFP fluorescence in OFF cells that were once ON was slightly above the autofluorescence measured in cells that did not express YFP. Therefore we used the YFP distribution from OFF cells that were once ON as the kernel. For cells that were once ON for CFP and have switched OFF, the level of CFP fluorescence was comparable to auto fluorescence. Then, we took the mean of the autofluorescence control as the OFF (zero) expression value. A portion of the measured fluorescence distribution mass falls below this value; we added this back to the first bin. Similarly, we took the mean of the ON peak as the value of μ/δ (maximum expression) and added back the portion of the fluorescence distribution mass above this value to the final bin.

Because of this operation, the resulting shape of the distribution is discontinuous between the first and second and penultimate and last bins. However, in most cases, this distribution could be fit directly to the beta distribution. Alternatively, the distribution could be further smoothed using a simple moving average prior to fitting to the model distribution. Both methods yield very similar fits.

We find that the values of the fit parameters λ and γ generally change 15-20% when the maximum expression value (μ/δ) selected is changed within a standard deviation about the mean of the ON peak. If we smooth the distribution using a moving average prior to fitting, the values of λ and γ could also change depending on the size of the spanning window used. For a bimodal distribution, the effect of increasing the span size is generally to increase both λ and γ, as the mass is distributed along a wider range, creating less distinct ON/OFF peaks. We therefore selected a small span sizes large enough to smooth jagged regions and always kept the span size uniform when comparing parameters between different regulator titrations.

In general, we have the best confidence in fitting bimodal distributions because we have an estimate of the maximum expression value from the ON peak. Unimodal ON distributions can be fit to a relatively wide range of high λ values and low to high γ values. For unimodal OFF distributions, it is difficult to estimate the maximum expression value. In titrations where the distributions were unimodal OFF (at low activator or high repressor levels) and then became bimodal at particular regulator levels, we set the value of μ/δ using the mean of the ON peak when in the bimodal regime. However, for regulator titrations of Tec1p, Ste12p or Phd1p in SD ura-, where distributions remained unimodal OFF at the highest activator levels, we chose the maximum fluorescence level as μ/δ. This choice of μ/δ represents a lower bound; the true maximum expression is likely higher. Fitting the same unimodal OFF distribution to a higher μ/δ will either decrease λ, increase γ, or both. Regardless, the estimated values of λ and γ for unimodal distributions no longer represent either fast or slow transition rates (see main text and below).

All fluorescence distributions were processed and the parameters λ and γ fit by maximum likelihood estimation using custom MATLAB routines.

**Transition rates in epigenetic versus conventional regulation**

When the two-state model (described by the Beta distribution) is used to derive OFF-ON transition rates, it is important to keep in mind that the underlying promoter states that we refer to as “OFF” or “ON” in the model are *different* depending on whether silencing occurs (Figure 4). As mentioned in the main text, in the case of the epigenetically regulated (silenced) promoter, the “OFF” to “ON” transition refers to the slow silenced to competent state transition. Because of the separation of timescales, the faster competent OFF to competent ACTIVE transition rates can all be lumped into a single, overall rate μ. Since the main source of variation in expression in an epigenetically regulated (partially silenced) promoter is the slow silenced to competent state transition, we can fit the static distributions and estimate λ and γ according to the procedure described in the previous section.

When the promoter is no longer silenced, only the fast promoter fluctuations between the competent OFF and competent ACTIVE states remain and cannot be neglected. This is now conventional regulation, where the variation in expression arises from faster extrinsic and intrinsic fluctuations. To measure the fast promoter transition rates *λ’* and *γ’*, we cannot use the fitting procedure described above, as the fit rates are no longer accurate since extrinsic fluctuations are significant. We must instead analyze the intrinsic noise using the method of [7]. In many eukaryotes, including yeast, these fast promoter fluctuations occur as bursts. Promoters primarily stay in the competent state, and make infrequent, random transitions to the active state, which correspond to a burst of transcription [8]. In other words, the active state is highly unstable. Global studies in yeast indicate that *trans* regulators control the level of gene expression by modulating the burst frequency, *λ’*, and not the burst size *μ’*/*γ’* [9, 10].

Figure 4 shows the regimes where true transition rates in epigenetically regulated and conventionally regulated genes lie. For epigenetic regulation, these are the rates of switching between the silent to competent OFF states, whereas for conventional regulation, these are the rates of switching between the competent OFF to competent ACTIVE states. Global studies of noise in yeast gene expression estimate a burst size of ~1200 proteins [9, 10].

In addition, we observed that the intrinsic noise in a *sfl1Δ* strain was unusually high, about ten times the intrinsic noise measured at *PHO84* (data not shown), whose high expression is comparable to *FLO11* in a *sfl1Δ*  background. The high intrinsic noise suggests the occurrence of a slow event (within a timescale of a cell division) prior to activation. Such an event might be chromatin remodeling to expose activator binding sites. If so, this could explain why much of the promoter remains nucleosome occluded in a *sfl1Δ* background (Figure S9). It remains an open question whether unusually high intrinsic noise might be a general property of heterochromatically silenced promoters when in a competent state, perhaps as a consequence of a particular nucleosomal structure that enables silencing.

**Determining fraction of “side populations” in static distributions using estimated false positive rate**

Cells expressing long-lived reporters at high levels require 3-5 divisions before fluorescence returns to autofluorescence levels and they actually appear OFF. The OFF state lifetime was ~3 cell division times on average in the time lapse experiments. Therefore, in a population of switching cells a fraction of cells that appear ON (above autofluorescence values) are actually OFF, resulting in a significant false positive rate. The false negative rate is negligible in comparison, because timelapse microscopy indicates that when OFF cells switch ON, fluorescence levels exceed autofluorescence significantly in much less than 1 cell division time.

We found good agreement with the values of λ and γ from fitting the static distributions to the stochastic kinetic model and the switching rates measured in the timelapse experiments. Therefore, we can apply this model to relate the true value of , the fraction of cells ON for a particular reporter, to the measured value , the *apparent* fraction of ON cells. From our two-state model :

$p=\frac{\lambda}{\lambda+\delta}$ (7)

The value is based on an arbitrary fluorescence threshold slightly above autofluorescence, chosen to be 3-5 standard deviations from the mean of the autofluorescence distribution, where the separation between the four states (both ON, both OFF, single CFP ON and single YFP ON) is well defined.

One can now define an estimate of the false positive rate, *x*, which is the fraction of cells that appear ON for a particular reporter but are really OFF. Then *x* can be simply calculated by

$x=1-\frac{p}{p'}$ (8)

where *p’* is obtained by direct measurement and *p* is estimated by fitting the fluorescence distribution to the stochastic model.

In the case of two reporters, the fraction of cells *apparently* ON for one reporter and not the other (“single ON”) is:

*apparent* “single ON” fraction (one particular reporter) =$p'(1-p')$ (9)

The actual “single ON” fraction consists of those cells that both appear to be and are “single ON” plus those cells that appear to be “both ON” are really “single ON” for a particular reporter:

*actual* single ON fraction =$(1-x)p'(1-p')+xp'(1-x)p'$ (10)

If both reporters are independent, then the total “side populations” is just twice equation (10). It is easy to verify that this is equivalent to$2p(1-p)$, where is the true fraction of ON cells, as shown in Figure 3D. We found that *x* was consistent across many conditions, and ranged between 0.30 and 0.36.

In all the regulator titrations and media conditions we have looked at, we have never observed well-separated subpopulations (both ON/OFF/ side populations) where the true fraction of ON cells > 0.5. It appears that around this point (perhaps at the critical γ?), the OFF-ON switching rate (λ) becomes much faster than 2 cell divisions, blurring the separation between states and making the fractions of observed side populations extremely sensitive to the value of the threshold fluorescence assigned. Therefore, we analyzed only the well-separated populations in all our experiments, where the true fraction of ON cells ranged from 0.03 to 0.4.

***FLO11* activation model predictions**

Our model of the kinetic roles of regulators makes several predictions about the nature of *FLO11* expression. Whereas a *sfl1Δ* expressed *FLO11* at high levels, an *sfl1Δflo8Δ* strain is reported to be OFF ^[11]^. We predict the promoter is in a competent state, and titration of either Class I or II activators will turn expression ON in a graded manner. Furthermore, the *FLO11* promoter should be hyperacetylated.

A second prediction is that the synthetic activator mimicking the Flo8p titration in SD ura- is capable of eliminating silencing at the promoter, even at low levels when expression is still OFF and the expression state is in the lower right quadrant (Figure 4C and 4D). Therefore constitutively expressing low levels of the synthetic activator in SD ura- media in the *absence* of ethanol should result in a competent promoter, which could be confirmed by ChIP for acetylation state and the titration of Class I activators.

**Binding site locations at *FLO11* promoter**

The location of binding sites shown in Figure 1 represent approximate positions only, as most sites were identified through a lacZ reporter assay where a fragment of the *FLO11* promoter was tested for its ability to activate expression of a lacZ reporter in various strain backgrounds. Therefore, the binding site(s) for each regulator lie within the range of the promoter fragment length used in the particular assay.

Ste12p and Tec1p binding sites were from [12], where 400bp FLO11 promoter fragments were used in a lacZ assay comparing activation in a wildtype and *trans* regulator delete strain. Binding sites for Tec1p and Ste12p were also found at -699 to -704 and -719 to -725 [13]; the consensus sequences (CATTC for Tec1p and TGAAAC for Ste12p) were originally identified using lacZ assays [14].

Using 250 bp promoter fragments, lacZ reporter assays and ChIP assays identified that Flo8p and Sfl1p both act upon the -1150 to -1400 region [11]; other Flo8p binding sites shown were determined from the lacZ assay of ^[12]^, which used 400 bp fragments.

The consensus sequence (CATGCA) for Phd1p has been identified [15] using a microarray-based technique; this sequence is found at -1763 and -1775.

**SUPPLEMENTARY REFERENCES**

1. Lorenz MC, Heitman J. (1997) Yeast pseudohyphal growth is regulated by GPA2, a G protein alpha homolog. EMBO J 16(23): 7008-7018.

2. Gietz RD, Woods RA. (2002) Transformation of yeast by lithium acetate/single-stranded carrier DNA/polyethylene glycol method. Methods Enzymol 350: 87-96.

3. Chen H, Fink GR. (2006) Feedback control of morphogenesis in fungi by aromatic alcohols. Genes Dev 20(9): 1150-1161.

4. Guo B, Styles CA, Feng Q, Fink GR. (2000) A saccharomyces gene family involved in invasive growth, cell-cell adhesion, and mating. Proc Natl Acad Sci U S A 97(22): 12158-12163.

5. Kaufmann BB, Yang Q, Mettetal JT, van Oudenaarden A. (2007) Heritable stochastic switching revealed by single-cell genealogy. PLoS Biol 5(9): e239.

6. Raj A, Peskin CS, Tranchina D, Vargas DY, Tyagi S. (2006) Stochastic mRNA synthesis in mammalian cells. PLoS Biology 4(10): e309 OP.

7. Elowitz MB, Levine AJ, Siggia ED, Swain PS. (2002) Stochastic gene expression in a single cell. Science 297(5584): 1183.

8. Maheshri N, O'Shea EK. (2007) Living with noisy genes: How cells function reliably with inherent variability in gene expression. Annu Rev Biophys Biomol Struct 36: 413-434.

9. Bar-Even A, Paulsson J, Maheshri N, Carmi M, O'shea E, et al. (2006) Noise in protein expression scales with natural protein abundance. Nat Genet 38(6): 636-643.

10. Newman JR, Ghaemmaghami S, Ihmels J, Breslow DK, Noble M, et al. (2006) Single-cell proteomic analysis of S. cerevisiae reveals the architecture of biological noise. Nature 441(7095): 840-846.

11. Pan X, Heitman J. (2002) Protein kinase A operates a molecular switch that governs yeast pseudohyphal differentiation. Mol Cell Biol 22(12): 3981-3993.

12. Rupp S, Summers E, Lo HJ, Madhani H, Fink G. (1999) MAP kinase and cAMP filamentation signaling pathways converge on the unusually large promoter of the yeast FLO11 gene. EMBO J 18(5): 1257-1269.

13. Lo WS, Dranginis AM. (1998) The cell surface flocculin Flo11 is required for pseudohyphae formation and invasion by saccharomyces cerevisiae. Mol Biol Cell 9(1): 161-171.

14. Madhani HD, Fink GR. (1997) Combinatorial control required for the specificity of yeast MAPK signaling. Science 275(5304): 1314-1317.

15. Badis G, Chan ET, van Bakel H, Pena-Castillo L, Tillo D, et al. (2008) A library of yeast transcription factor motifs reveals a widespread function for Rsc3 in targeting nucleosome exclusion at promoters. Mol Cell 32(6): 878-887.

16. Whitehouse I, Rando OJ, Delrow J, Tsukiyama T. (2007) Chromatin remodelling at promoters suppresses antisense transcription. Nature 450(7172): 1031-1035.

17. Kaplan N, Moore IK, Fondufe-Mittendorf Y, Gossett AJ, Tillo D, et al. (2008) The DNA-encoded nucleosome organization of a eukaryotic genome. Nature 458(7236): 362-366.

18. Mavrich TN, Ioshikhes IP, Venters BJ, Jiang C, Tomsho LP, et al. (2008) A barrier nucleosome model for statistical positioning of nucleosomes throughout the yeast genome. Genome Res 18(7): 1073-1083.
